# Heat Shock-Induced Accumulation of Translation Elongation and Termination Factors Precedes Assembly of Stress Granules in *S. cerevisiae*

**DOI:** 10.1371/journal.pone.0057083

**Published:** 2013-02-25

**Authors:** Tomas Grousl, Pavel Ivanov, Ivana Malcova, Petr Pompach, Ivana Frydlova, Renata Slaba, Lenka Senohrabkova, Lenka Novakova, Jiri Hasek

**Affiliations:** Institute of Microbiology of AS CR, v.v.i., Prague, Czech Republic; University of Kent, United Kingdom

## Abstract

In response to severe environmental stresses eukaryotic cells shut down translation and accumulate components of the translational machinery in stress granules (SGs). Since they contain mainly mRNA, translation initiation factors and 40S ribosomal subunits, they have been referred to as dominant accumulations of stalled translation preinitiation complexes. Here we present evidence that the robust heat shock-induced SGs of *S. cerevisiae* also contain translation elongation factors eEF3 (Yef3p) and eEF1Bγ2 (Tef4p) as well as translation termination factors eRF1 (Sup45p) and eRF3 (Sup35p). Despite the presence of the yeast prion protein Sup35 in heat shock-induced SGs, we found out that its prion-like domain is not involved in the SGs assembly. Factors eEF3, eEF1Bγ2 and eRF1 were accumulated and co-localized with Dcp2 foci even upon a milder heat shock at 42°C independently of P-bodies scaffolding proteins. We also show that eEF3 accumulations at 42°C determine sites of the genuine SGs assembly at 46°C. We suggest that identification of translation elongation and termination factors in SGs might help to understand the mechanism of the eIF2α factor phosphorylation-independent repression of translation and SGs assembly.

## Introduction

Reactivity to environmental changes is a key property of all living organisms that enables them to survive and develop. Cells undergo adaptation to stress conditions via various mechanisms. To save energy, general translation is reduced and the expression of stress response-specific genes is triggered. New ribonucleoprotein (RNP) complexes are formed, through which the fate of mRNA molecules and translation machinery components is regulated.

Major accumulations of cytoplasmic RNP complexes that have been recognized in higher eukaryotes - processing bodies (P-bodies) and stress granules (SGs) have also been found in yeasts [1], [2], [3], [4], [5], [6]. Whilst P-bodies are present even in unstressed cells and they become dominant upon stress, SGs are formed only in stressed cells. Besides translationally repressed mRNA molecules, P-bodies also contain proteins involved in mRNA degradation, translation repression, mRNA quality control and other functions (for a review, see [7], [8]). However, nowadays it is evident that the function of P-bodies is far more complex than merely mRNA degradation [9], [10], [11], [12] and is still not well understood (for a review, see [13]). The composition of SGs is influenced by the particular stress conditions and depends on the organism being subjected to the stress. It is generally accepted that major SGs components are stalled translation preinitiation complexes (48S), which contain molecules of mRNA, small ribosomal subunits and several translation initiation factors. Besides these, they may contain a few other factors, which could be found either in SGs or P-bodies [2], [14]. Thus, P-bodies and SGs represent two distinct types of RNP assemblies, which can share some components and can be in spatial contact, but differ in their role in cell adaptation to environmental changes, which is realized via translation regulation and mRNA metabolism.

Analogous to mammalian cells, it is thought that yeast SGs take part in regulation of translation, sorting and storage of mRNA molecules, and the preservation of selected translation factors and mRNA molecules against an influence of a stress [10], [14], [15]. Recently, they have been shown to control TORC1 signaling [16]. Typical examples of yeast SGs are those containing eIF3 components. They are induced by robust heat shock [2], high concentrations of ethanol [3] or NaN_3_ [17]. However, only the robust heat shock-induced SGs accumulate 40S ribosomal subunits. Based on the analyses of the strains from the GFP collection [18], none of the tested 60S markers formed foci upon robust heat shock indicating that 60S ribosomal subunits were not accumulated in these SGs [2]. Interestingly, these typical SGs containing 40S and eIF3 translation initiation factor have also been observed in fission yeast *S. pombe* affected by heat shock or glucose deprivation [4], [19]. Other examples of RNP accumulations, which are thought to be functionally analogous to SGs, are yeast EGP bodies [20], stress granules of glucose-deprived [1] or cold-stressed [6] *S. cerevisiae* cells.

Heat stress is an example of stress conditions resulting in the formation of either P-bodies or stress granules (SGs). In *S. cerevisiae* cells, whose temperature optimum for growth is between 25°C and 30°C, cultivation at 39°C triggers the assembly of P-bodies [21] and robust heat shock at 46°C leads to the formation of SGs [2]. Here we report on the identification of novel components of heat-induced SGs in yeast, translation elongation factors eEF3 (Yef3p) and eEF1Bγ2 (Tef4p) and translation termination factors eRF1 (Sup45p) and eRF3 (Sup35p). Despite the presence of the yeast prion protein Sup35, we proved that its prion-like domain is necessary neither for SGs assembly nor for its localization into SGs. Moreover, we showed that eEF3, eEF1Bγ2 and eRF1 factors accumulate even upon a milder heat shock at 42°C, possibly preparing a platform for assembly of genuine SGs at 46°C. Although RNP accumulations induced at 42°C contain some components of P-bodies, they depend neither on P-bodies scaffolding proteins nor on the Gcn2 kinase activity.

The Gcn2 kinase is so far the only known yeast kinase of α subunit of translation initiation factor 2 (eIF2α) [22]. This factor plays an essential role in translation initiation and its regulation. The eIF2 factor is a component of the “ternary complex”, which brings the initiator tRNA to 40S ribosomal subunits. The GTP-bound to eIF2 is then hydrolyzed to GDP and the factor leaves the ribosome. The active, GTP-bound, form of eIF2 is recovered by its guanine nucleotide exchange factor eIF2B [23]. Under stress conditions, stress responsive kinases, e.g. Gcn2, phosphorylate α subunit of eIF2 factor [22]. Since the phosphorylated eIF2 factor has higher affinity for eIF2B [24], it leads to reduction of availability of eIF2B factor, eIF2-GTP and the “ternary complex”. Thus, it results in inhibition of translation initiation, which is commonly link to an accumulation of stalled translation preinitiation complexes and stress granules assembly. Taken together, the phosphorylation of eIF2α factor is sufficient, but not necessary for SGs formation (for a review, see [14]). The eIF2α-phosphorylation independent mechanisms of SGs assembly have also been described from yeast to mammalian cells [2], [25], [26], [27], [28], [29], [30]. These mechanisms are mainly concerned with translation initiation factors, or are yet unknown [31].

Although, we found that assembly of heat-induced SGs at 46°C and RNP accumulations formed at 42°C are not driven by eIF2α phosphorylations, we also observed that the translation initiation factor eIF2α (Sui2p), which is not a component of the heat-induced SGs in *S. cerevisiae*, accumulates when SGs dissolve during the cell recovery from the stress. Altogether, our data might help to understand the eIF2α factor phosphorylation-independent signaling pathway of the SGs assembly and to reinforce the hypothesis that SGs are the sites where translation initiates after stress relief.

## Materials and Methods

### Yeast Strains and Growth Conditions

The *Saccharomyces cerevisiae* strains used in this study were derived either from the BY [32], S288C [18] or SEY6210 [33] backgrounds and are listed in Table 1. Yeast cultures were grown in YPD medium (1% yeast extract, 2% peptone, 2% glucose) or SC medium (0.17% YNB without amino acids and ammonium sulfate, 0.5% ammonium sulfate, 2% glucose, supplemented with a complete or appropriate mixture of amino acids) at 30°C. The corresponding solid media contained 2% agar. To select for auxotrophies, the respective amino acid was omitted from the dropout mix. To select for resistance to antibiotics, the appropriate antibiotic was added to the media. Standard methods were used for all DNA manipulations [34]. Strains and mutants expressing particular combinations of various GFP/RFP/mCherry fusion proteins from the sites on the chromosomes were generated by mating, sporulation on solid Fowell medium and subsequent spore dissection by Singer™ micromanipulator. To perform the heat shock, cells were re-suspended in YPD medium preheated to 42°C or 46°C and incubated under shaking at the given temperature for additional 10 minutes. Cycloheximide (Sigma-Aldrich, USA) was added to the final concentration of 50 *µ*g/ml when appropriate.

**Table 1:** Yeast strains.

Strain	Genotype	Source
BY4741	*MAT*a *his3Δ1 leu2Δ0 met15Δ0 ura3Δ0*	[32]
BY4742	*MAT*α *his3Δ1 leu2Δ0 lys2Δ0 ura3Δ0*	[32]
BY4743	*MAT* a/α *his3Δ1/his3Δ1 leu2Δ0/leu2Δ0 lys2Δ0*/*LYS2 MET15*/*met15Δ0 ura3Δ0/ura3Δ0*	[32]
S288C	*MAT*α *SUC2 gal2 mal mel flo1 flo8-1 hap1 ho bio1 bio6*	[56]
Sey6210	*MAT*α *leu2-3, 112 ura3-52 his3-Δ200 trp1-Δ901 lys2-801 suc2-Δ9*	[33]
Sey6210.1	*MAT*a *leu2-3,112 ura3-52 his3-Δ200 trp1-Δ901 lys2-801 suc2-Δ9*	[33]
CRY255	Sey6210; *MAT*α *RPG1-RFP::kan*MX4	[2]
CRY309	BY4741; *MAT*a *gcn2::kan*MX4	EUROSCARF
CRY412	S288C; *MAT*a *SUP35-GFP::HIS3*MX6	[18]
CRY423	S288C; *MAT*a *PAB1-GFP::HIS3*MX6	[18]
CRY437	S288C; *MAT*a *SUI2-GFP::HIS3*MX6	[18]
CRY462	S288C; *MAT*a *NGR1-GFP::HIS3*MX6	[18]
CRY510	S288C; *MAT*a *YEF3-GFP::HIS3MX6*	[18]
CRY521	S288C; *MAT*a *TIF11-GFP::HIS3MX6*	[18]
CRY528	Sey6210 × S288C; *MAT*α *RPG1-RFP::kan*MX4 *PAB1-GFP::HIS3*MX6	This study
CRY552	S288C; *MAT*a *EFT1-GFP::HIS3*MX6	[18]
CRY554	S288C; *MAT*a *TEF4-GFP::HIS3*MX6	[18]
CRY564	Sey6210 × S288C; *MAT*a *RPG1-RFP::kan*MX4 *DCP2-GFP::HIS3*MX6	[2]
CRY582	S288C × BY4742; *MAT*α *gcn2::kan*MX4	This study
CRY649	Sey6210 × S288C; *MAT*a *RPG1RFP::kan*MX4 *YEF3-GFP::HIS3*MX6	This study
CRY977	Sey6210 × S288C; *MAT*a *RPG1-RFP::kan*MX4 *DCP2-GFP::HIS3*MX6 *edc3::URA3 lsm4ΔC::LEU2*	[2]
CRY993	BY4743; *MAT*a/α *sup35::kan*MX4*/SUP35*	EUROSCARF
CRY998	BY4741 × BY4742; *MAT*α *sup35::kan*MX4+ pLewi0564 (Ura+)	This study
CRY1001	BY4741 × BY4742; *MAT*α *sup35::kan*MX4+ pLewi0512 (Ura+)	This study
CRY1007	BY4741 × BY4742 × S288C; *MAT*α *sup35::kan*MX4 *RPG1-GFP::HIS3*MX6+ pLewi0512 (Ura+)	This study
CRY1011	BY4741 × BY4742 × S288C; *MAT*a *sup35::kan*MX4 *RPG1-GFP::HIS3*MX6+ pLewi0564 (Ura+)	This study
CRY1035	Sey6210 × S288C; *MAT*a *RPG1-RFP::kan*MX4 *PUB1-GFP::HIS3*MX6 *edc3::URA3 lsm4ΔC::LEU2*	This study
CRY1041	Sey6210 × S288C; *MAT*α *RPG1-RFP::kan*MX4 *NGR1-GFP::HIS3*MX6 *edc3::URA3 lsm4ΔC::LEU2*	This study
CRY1043	BY4742; *MAT*α *edc3::URA3 lsm4ΔC::LEU2*	This study
CRY1146	S288C × BY4742; *MAT*α *YEF3-GFP::HIS3*MX6 *edc3::URA3 lsm4ΔC::LEU2*	This study
CRY1287	S288C; *MAT*a *YEF3-mCherry::nat*NT2 *NGR1-GFP::HIS3*MX6	This study
CRY1288	BY4742; *MAT*α *DCP2-mCherry:: nat*NT2	This study
CRY1289	BY4742; *MAT*α *NGR1-mCherry:: nat*NT2	This study
CRY1292	S288C × BY4742; *MAT*a *gcn2::kanMX YEF3-GFP::HIS3*MX6	This study
CRY1315	BY4742 × S288C; *MAT*a *YEF3-mCherry:: nat*NT2 *TEF4-GFP::HIS3*MX6	This study
CRY1332	Sey6210 × S288C; *MAT*a *RPG1-RFP::Kan*MX4 *SUI2-GFP::HIS3*MX6	This study
CRY1336	S288C × BY4742; *MAT*α *YEF3-GFP::HIS3*MX6 *gcn2::kan*MX4	This study
CRY1339	BY4742; *MAT*α *YEF3-mCherry:: nat*NT2	This study
CRY1364	Sey6210 × S288C; *MAT*a *RPG1-RFP::kan*MX4 *SUP35-GFP::HIS3*MX6	This study
CRY1516	S288C; *MAT*a *STM1-GFP::HIS3*MX6	[18]
CRY1552	S288C; *MAT*a *SUP45-GFP::HIS3*MX6	[18]
CRY1559	BY4742 × S288C; *MAT*α *YEF3-mCherry:: nat*NT2 *DCP2-GFP::HIS3*MX6	This study
CRY1618	BY4742 × Sey6210.1; *MAT*α *YEF3-mCherry:: nat*NT2+ pRP1187 (Trp+)+pPS2037 (Ura+)	This study
CRY1627	Sey6210 × S288C; *MAT*a/α *RPG1-RFP::kan*MX4 *SUP45-GFP::HIS3*MX6	This study
CRY1636	BY4742 × S288C; *MAT*α *DCP2-mCherry:: nat*NT2 *SUP45-GFP::HIS3*MX6	This study
CRY1638	BY4742 × S288C; *MAT*α *NGR1-mCherry:: nat*NT2 *SUP45-GFP::HIS3*MX6	This study
CRY1691	Sey6210 × S288C × BY4742; *MAT*a *RPG1-RFP::kan*MX4 *SUI2-GFP::HIS3*MX6 *gcn2::kan*MX4	This study
CRY1704	BY4741; *MAT*a *gcn2::kan*MX4	EUROSCARF
CRY1760	Sey6210 × S288C × BY4741; *MAT*a *RPG1-RFP::kan*MX4 *PAB1-GFP::HIS3*MX6 *stm1::kan*MX4	This study
CRY1764	S288C; *MAT*a *TEF1-GFP::HIS3*MX6	[18]

### Plasmids

Plasmids pRP2037 and pRP1187 for *PGK1* mRNA localization by U1A-GFP fusion protein [5], [9] were kindly provided by R. Parker (University of Arizona, USA). For generation of strains expressing only a C-terminal part of Sup35 protein and wild-type form of Sup35 protein as a control, plasmids pLewi0512 and pLewi0564 were used (a kind gift from E.I. Lewitin, Institute of Industrial Genetics, Russia).

*Escherichia coli* strain DH5α [F^-^ *rec A1 supE44 endA1 hsdR17 (rk-, mk+) gyrA96 relA1 thi-1 Δ(lacIZYA-argF)U169 deoR (Φ80dΔ(lacZ*) *M15*] [35] was cultivated in LB medium (1% trypton, 0.5% yeast extract and 1% NaCl) and used for plasmids propagation.

### Construction of Strains with Chromosome-derived Expression of mCherry Fusions

To create C-terminal genomic fusions of genes of interest, the mCherry integrative cassette containing mCherry fluorescent protein and the selection marker *nat*NT2 (nourseothricin) was used. The cassette was amplified using ORF-specific primers by PCR on the template plasmid pFM699 (kindly provided by M. Farkasovsky, Slovak Academy of Sciences, Slovakia). Purified PCR products were transformed into the appropriate cells and transformants were selected on YPD plates containing 100 *µ*g/ml of nourseothricin (WERNER BioAgents, Germany). Correct integration of the cassette was confirmed by PCR, fluorescence microscopy and Western blotting.

### Construction of *edc3Δlsm4ΔC* Mutant Strain

The mutant strain *edc3*Δ*lsm4*ΔC was constructed by a repeated one-step gene disruption technique [36]. The deletion cassettes loxP-*URA3*-loxP and loxP-*LEU2*-loxP were amplified from pUG72 and pUG73 respectively [37], using the ORF-specific primers. The mutant *lsm4*ΔC denotes a partial deletion of 97 C-terminal amino acids of Lsm4 protein [8].

### Polysome Profile Analyses

Cells were grown to an OD_600_ ∼1 and cycloheximide (CYH; Sigma-Aldrich, USA) was added to the culture to a final concentration of 50 *µ*g/ml 5 min before harvesting. Chilled cells were washed in the GA buffer (20 mM Tris-HCl pH 7.5, 50 mM KCl, 10 mM MgCl_2_, 1 mM DTT, and 5 mM NaF) containing 50 *µ*g/ml CYH. Lysates were prepared in the GA buffer (supplemented with 1 tablet of Complete Mini EDTA-free Protease Inhibitor Mix (Roche, Switzerland)/10 ml of the buffer and 50 *µ*g/ml of cycloheximide) using glass beads in Fastprep Bio101 (Savant Instruments, USA) at speed 5 for 20 s. Lysates were precleared by centrifugation at 826 × *g* for 5 min and at 13,224 × *g* for 10 min, and loaded on a 5–45% sucrose gradient. Gradients were ultracentrifuged at 260,500 × *g* at 4°C for 2.5 h. Fractions were collected from the top and A_254_ was recorded.

### Analyses of Protein Accumulations

Exponentially growing cells were heat-shocked at 46°C for 10 min and harvested. Cells were washed and resuspended in the lysis buffer [9] containing 50 mM Tris-HCl (pH 7.6), 50 mM NaCl, 5 mM MgCl_2_, 0.1% NP-40, 1 mM β-mercaptoethanol and 1 tablet of Complete Mini EDTA-free Protease Inhibitor Mix (Roche, Switzerland)/10 ml of the buffer. The disruption of cells was carried out twice in a Fastprep Bio101 (Savant Instruments, USA) at speed 4 for 20 s. Cell debris were pelleted at 2,296 × *g* at 4°C for 10 minutes. The supernatant was centrifuged at 18,000 × *g* at 4°C for 10 minutes. Resulting pellets and supernatants were analyzed by SDS-PAGE and Western blotting for the presence of GFP-tagged fusion proteins.

### Western Blot Analyses

Proteins resolved by SDS-PAGE were transferred to a Protran nitrocellulose membrane (Sigma-Aldrich, USA). The membrane blots were blocked with 3% non-fat milk in TBS-T buffer and incubated overnight with appropriate antibodies. Mouse monoclonal anti-GFP HRP-conjugated antibody (Santa Cruz Biotechnology, USA) was used at 1∶2000. Rabbit polyclonal anti-Sup35 antibody was used at 1∶2000. Donkey anti-rabbit IgG antibody conjugated with a horseradish peroxidase (GE Healthcare; England) was used as a secondary antibody.

### Microscopy

The cells were inspected after washing with SC medium, mounting on coverslips and coating with a slice of 1.5% agarose in an appropriate medium. The distribution of various fusion proteins (fused to GFP or RFP or mCherry) was analyzed with a 100x PlanApochromat objective (NA = 1.4) using an Olympus IX-81 inverted microscope equipped with a Hammamatsu Orca/ER digital camera and an Olympus CellR™ detection and analyzing system (GFP filter block U-MGFPHQ, exc. max. 488, em. max. 507; RFP filter block U-MWIY2, exc. max. 545–580, em. max. 610). Images were processed and merged using Olympus CellR™ and Adobe CS5 software. The quantitative co-localization analyses were performed using NIH ImageJ software with the Co-localization Finder plugin, available at http://rsb.info.nih.gov/ij/plugins/. This software was used to determine the Pearson’s correlation coefficient (R_r_), which describes the extent of overlap between image pairs. It is a value between −1 and +1, with −1 being no overlap and +1 being perfect overlap of the two images. The time-lapse experiments were done using an ONIX microfluidic perfusion system (Cell Asic Corp., USA; http://www.cellasic.com).

### In-gel Digestion and MALDI-TOF Mass Spectrometry

The spots of interest were cut from the CBB-stained SDS–PAGE gels, destained by a mixture of 100 mM ethylmorpholine acetate buffer and acetonitrile (1∶1), and reduced with Tris(2-carboxyethyl)phosphine hydrochloride. Reduced cysteines were then alkylated with 50 mM iodoacetic acid. Gel pieces were washed three times with acetonitrile and water. Trypsin protease was added to the gel in the digestion buffer (50 mM ethylmorpholine acetate buffer, 10% acetonitrile, pH 8.3). After an overnight incubation, tryptic peptides were extracted from the gel by the addition of 80% acetonitrile, 0.1% trifluoroacetic acid.

Extracted peptides were desalted using a Peptide Microtrap in the off-line holder (MichromBioresources, USA). Each sample was spotted in one position of the 384-position ground steel MALDI plate (Bruker Daltonics, USA). α-Cyano-4-hydroxycinnamic acid was used as a matrix (Bruker Daltonics). Samples were ionized by matrix-assisted laser desorption ionization (MALDI) using a Dual II ion source (Bruker Daltonics). Mass spectra were acquired in an APEX-Qe Fourier transformation mass spectrometry instrument equipped with a 9.4 T superconducting magnet (Bruker Daltonics). The cell was opened for 4 ms; accumulation time was set to 0.2 s, and one experiment consisted of the average of four spectra. The acquisition data set size was set to 512,000 points, with the mass range starting at m/z 600 atomic mass units. The instrument was externally calibrated using Bruker Daltonics calibration standard II. The spectra were processed by Data Analysis 4.0 software (Bruker Daltonics) and searched with the Mascot search engine (http://www.matrixscience.com) against the data base (UniProt Knowledgebase; http://www.ebi.ac.uk/uniprot) created from all known *S. cerevisiae* proteins.

## Results

### Translation Elongation Factor eEF3 (Yef3p) is a Novel Component of Heat-induced SGs of *S. cerevisiae*

**Figure 1:**
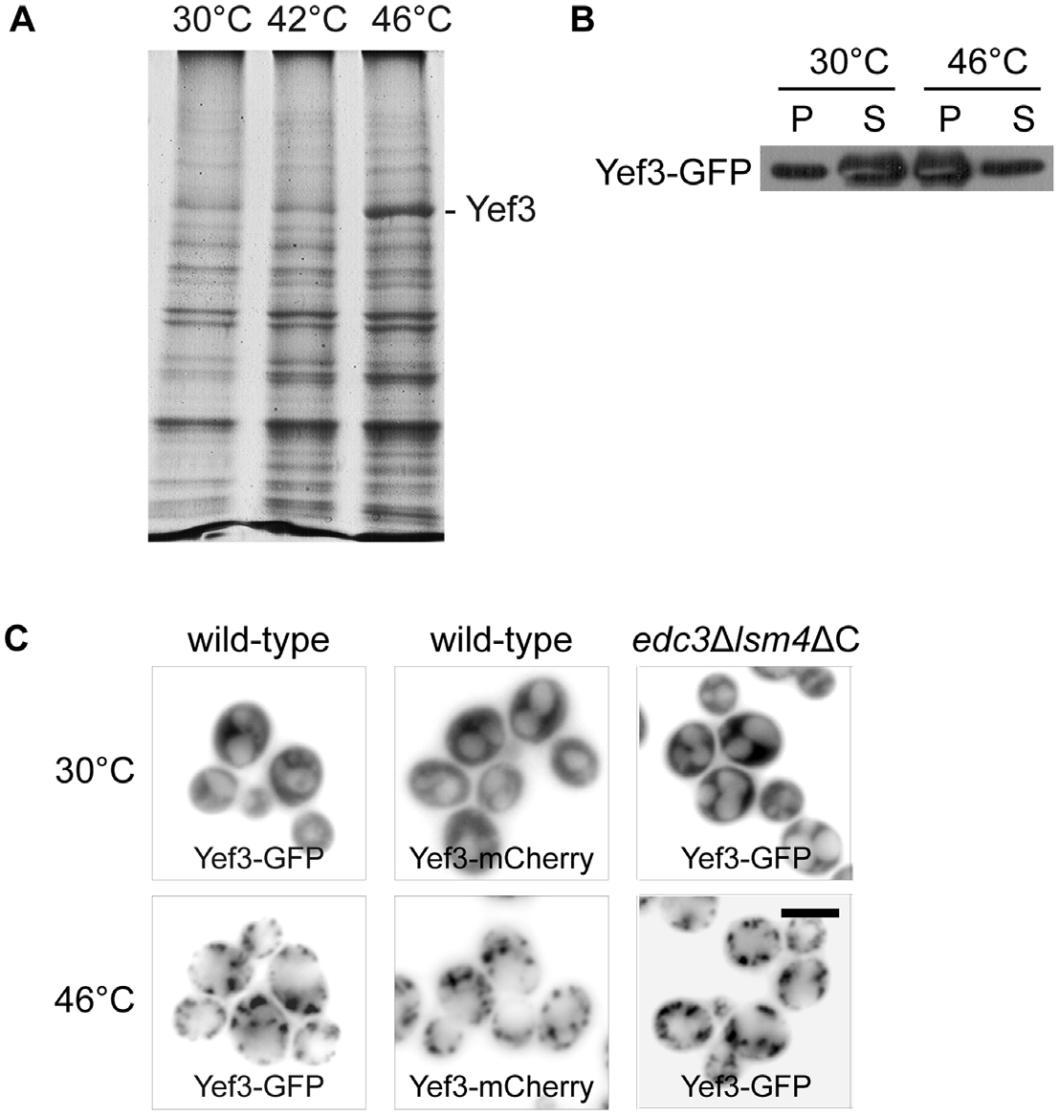
Translation elongation factor eEF3 (Yef3p) accumulates in foci upon robust heat shock. (A) Pellet fractions from the *edc3*Δ*lsm4*ΔC cell (CRY1043 strain) lysates taken upon control (30°C) and heat shock (42°C and 46°C) conditions were separated by SDS-PAGE and stained with Coomassie Brilliant Blue. Yef3 protein, identified by MS analysis, is enriched in the pellet fraction from the cells heat-shocked at 46°C for 10 minutes. (B) Pellet and supernatant fractions from the lysates of cells expressing Yef3-GFP (CRY510 strain) were analyzed by SDS-PAGE and Western blotting. Samples were prepared as described elsewhere [2]. The Yef3-GFP fusion protein was accumulated in the pellet fraction of heat-shocked cells. (C) The Yef3 fusions were accumulated at cytoplasmic foci in wild-type cells (CRY510 and CRY1339 strains) as well as in the *edc3*Δ*lsm4*ΔC mutant cells (CRY1146 strain) heat-shocked at 46°C for 10 minutes. Scale bar 4 µm.

In our previous work, we described that formation of the robust heat shock-induced stress granules (SGs) is promoted by P-bodies in *S. cerevisiae* [2]. In addition, we also described that these SGs are formed even in the *edc3*Δ*lsm4*ΔC mutant, which is unable to form visible P-bodies under glucose deprivation [8]. Therefore, we used the *edc3*Δ*lsm4*ΔC mutant in our recent experiments to identify additional components of SGs in heat-shocked cells. We applied a biochemical approach combined with mass spectrometry analyses. We stressed the exponentially growing *edc3*Δ*lsm4*ΔC mutant cells either at 42°C or at 46°C for 10 minutes. The protein samples from the heat-shocked cells were prepared by differential centrifugation according to the protocol we used previously [2] and proteins in the pellet were separated by SDS-PAGE. A 116*-*kD protein appeared to accumulate predominantly in the pellet of the mutant cells heat-shocked at 46°C (Fig. 1A). The protein band was cut out of the gel and subjected to enzymatic digestion in suspension followed by the mass spectrometry analysis, which identified only peptides of the translation elongation factor eEF3 (Yef3p) (Table 2). This result was further confirmed by the Western blot analysis of pelleted proteins prepared by differential centrifugation of the lysate from the wild-type cells expressing Yef3-GFP (Fig. 1B). To perform microscopic analyses of heat-shocked cells, we used either the Yef3-GFP strain from the GFP collection [18] or a newly prepared Yef3-mCherry expressing strain. The distribution of both fusion proteins in wild-type cells was uniformly cytosolic at 30°C (Fig. 1C). They both were accumulated in cytoplasmic foci after heat-shock at 46°C for 10 minutes. In heat-shocked cells harboring the *edc3*Δ*lsm4*ΔC deletion and the Yef3-GFP fusion, we observed a similar pattern of the fluorescent foci-containing Yef3-GFP as found in wild-type cells (Fig. 1C).

**Table 2:** MS identification of Yef3 (eEF3) protein.

Identified peptides: sequence				start – end	
K.ALLPHLTNAIVETNK.W				128–142	
R.MPELIPVLSETMWDTKK.E				168–184	
K.ATETVDNKDIER.F				197–208	
K.LVEDPQVIAPFLGK.L				276–289	
K.SNFATIADPEAR.E				296–307	
K.IVVEYIAAIGADLIDER.I				360–376	
K.AKDILDEFR.K				400–408	
K.DILDEFR.K				402–408	
K.DILDEFRK.R				402–409	
R.YGICGPNGCGKSTLMR.A				459–474	
R.YGICGPNGCGKSTLMR.A				459–474	
R.TVYVEHDIDGTHSDTSVLDFVFESGVGTK.E				492–520	
K.AYEELSNTDLEFKFPEPGYLEGVK.T				637–660	
K.FPEPGYLEGVK.T				650–660	
K.QHAFAHIESHLDK.T				738–750	
K.TPSEYIQWR.F				751–759	
R.FQTGEDRETMDR.A				760–771	
R.IAGIHSR.R				797–803	
R.IAGIHSRR.K				797–804	
K.MVAEVDMKEALASGQFRPLTR.K				852–872	
K.EALASGQFRPLTR.K				860–872	
K.ALKEFEGGVIIITHSAEFTK.N				938–957	
K.EFEGGVIIITHSAEFTK.N				941–957	
K.LSSAELRK.K				1014–1021	
**ORF translation sequence of Yef3 protein:**					
**(matched peptides shown in bold)**					
MSDSQQSIKV	LEELFQKLSV	ATADNRHEIA	SEVASFLNGN		IIEHDVPEHF
FGELAKGIKD	KKTAANAMQA	VAHIANQSNL	SPSVEPYIVQ		LVPAICTNAG
NKDKEIQSVA	SETLISIVNA	VNPVAIK**ALL**	**PHLTNAIVET**		**NK**WQEKIAIL
AAISAMVDAA	KDQVALR**MPE**	**LIPVLSETMW**	**DTKK**EVKAAA		TAAMTK**ATET**
**VDNKDIER**FI	PSLIQCIADP	TEVPETVHLL	GATTFVAEVT		PATLSIMVPL
LSRGLNERET	GIKRKSAVII	DNMCK**LVEDP**	**QVIAPFLGK**L		LPGLK**SNFAT**
**IADPEAR**EVT	LRALKTLRRV	GNVGEDDAIP	EVSHAGDVST		TLQVVNELLK
DETVAPRFK**I**	**VVEYIAAIGA**	**DLIDER**IIDQ	QAWFTHITPY		MTIFLHEKK**A**
**KDILDEFRK**R	AVDNIPVGPN	FDDEEDEGED	LCNCEFSLAY		GAKILLNKTQ
LRLKRARR**YG**	**ICGPNGCGKS**	**TLMR**AIANGQ	VDGFPTQEEC		R**TVYVEHDID**
**GTHSDTSVLD**	**FVFESGVGTK**	EAIKDKLIEF	GFTDEMIAMP		ISALSGGWKM
KLALARAVLR	NADILLLDEP	TNHLDTVNVA	WLVNYLNTCG		ITSITISHDS
VFLDNVCEYI	INYEGLKLRK	YKGNFTEFVK	KCPAAK**AYEE**		**LSNTDLEFKF**
**PEPGYLEGVK**	TKQKAIVKVT	NMEFQYPGTS	KPQITDINFQ		CSLSSRIAVI
GPNGAGKSTL	INVLTGELLP	TSGEVYTHEN	CRIAYIK**QHA**		**FAHIESHLDK**
**TPSEYIQWRF**	**QTGEDRETMD**	**R**ANRQINEND	AEAMNKIFKI		EGTPRR**IAGI**
**HSRR**KFKNTY	EYECSFLLGE	NIGMKSERWV	PMMSVDNAWI		PRGELVESHS
K**MVAEVDMKE**	**ALASGQFRPL**	**TR**KEIEEHCS	MLGLDPEIVS		HSRIRGLSGG
QKVKLVLAAG	TWQRPHLIVL	DEPTNYLDRD	SLG**ALSKALK**		**EFEGGVIIIT**
**HSAEFTK**NLT	EEVWAVKDGR	MTPSGHNWVS	GQGAGPRIEK		KEDEEDKFDA
MGNKIAGGKK	KKK**LSSAELR**	**K**KKKERMKKK	KELGDAYVSS		DEEF

**Figure 2:**
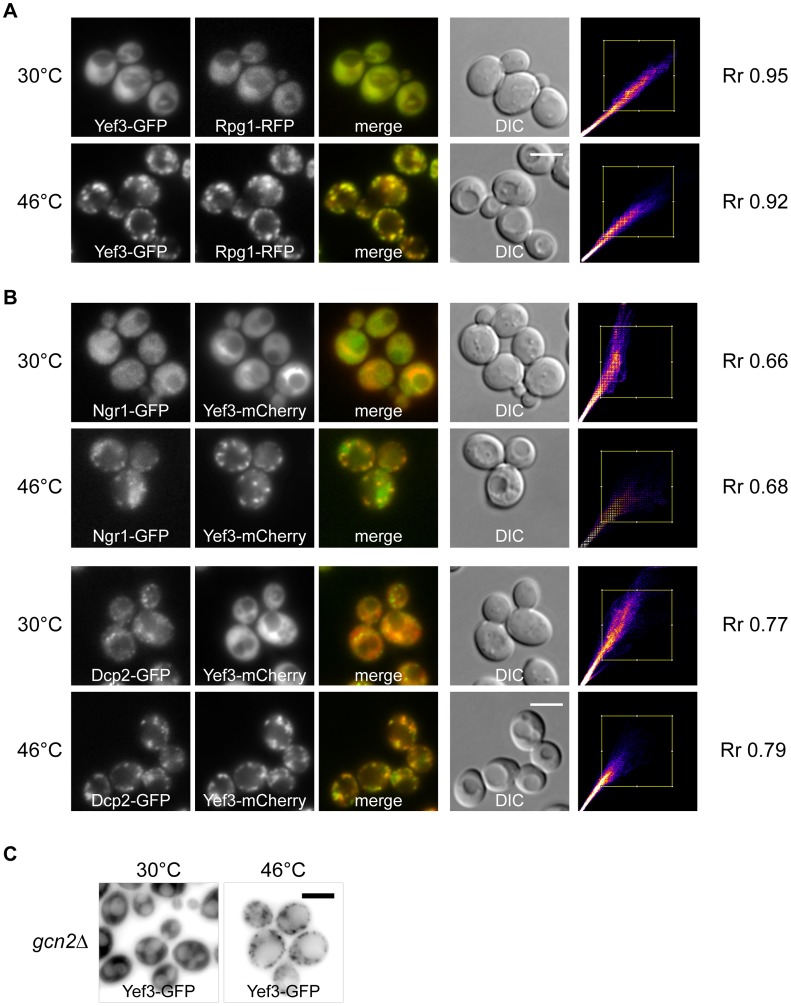
Heat shock-induced stress granules contain translation elongation factor eEF3 (Yef3p). (A) The distribution of fusion proteins Yef3-GFP and Rpg1-RFP (CRY649 strain) was uniformly cytosolic at 30°C. Accumulations of Yef3-GFP fusion protein completely co-localized with accumulations of the stress granule marker protein Rpg1-GFP in cells heat-shocked at 46°C for 10 minutes. The Scatter plot produced by colocalization analysis software shows colocalized pixels along the diagonal. Very high values of the Pearsońs correlation coefficient (R_r_), over 0.9, confirmed the high degree of this co-localization. (B) Localization of theYef3-mCherry fusion protein with Ngr1-GFP (CRY1287 strain) and Dcp2-GFP (CRY1559 strain) fusion proteins under control conditions (30°C) and in heat-shocked cells at 46°C for 10 minutes. Yef3-mCherry protein co-localized with heat-induced accumulations of stress granules marker proteins Dcp2-GFP and Ngr1-GFP. (C) Yef3-GFP accumulates in cytoplasmic foci in both, wild-type (CRY510 strain) and *gcn2*Δ (CRY1292 strain), cells heat-shocked at 46°C. Scale bar 4 µm.

To investigate whether these Yef3-containing foci were identical to previously identified heat-induced SGs, we prepared a new strain co-expressing Yef3-GFP together with the stress granule marker Rpg1-RFP. We observed that both proteins co-localized in SGs of heat-shocked cells (Fig. 2A). Very high values of the Pearsońs correlation coefficient (R_r_), over 0.9, confirmed the high degree of this co-localization. Similar results were obtained in experiments with the strains co-expressing Yef3-mCherry and other constituents of heat-induced SGs, Ngr1-GFP or Dcp2-GFP proteins (Fig. 2B). The presence of the Yef3 protein accumulations in heat-shocked cells of the *gcn2*Δ mutant indicated that their assembly is also independent of the eIF2α factor phosphorylation, as we reported previously for accumulations of eIF3a (Rpg1p/Tif32p) [2] (Fig. 2C). We conclude that the translation elongation factor eEF3 (Yef3p) is a novel component of the heat-induced SGs in *S. cerevisiae*.

### SGs also Contain Translation Factors eEF1Bγ2 (Tef4p), eRF1 (Sup45p) and eRF3 (Sup35p)

**Figure 3:**
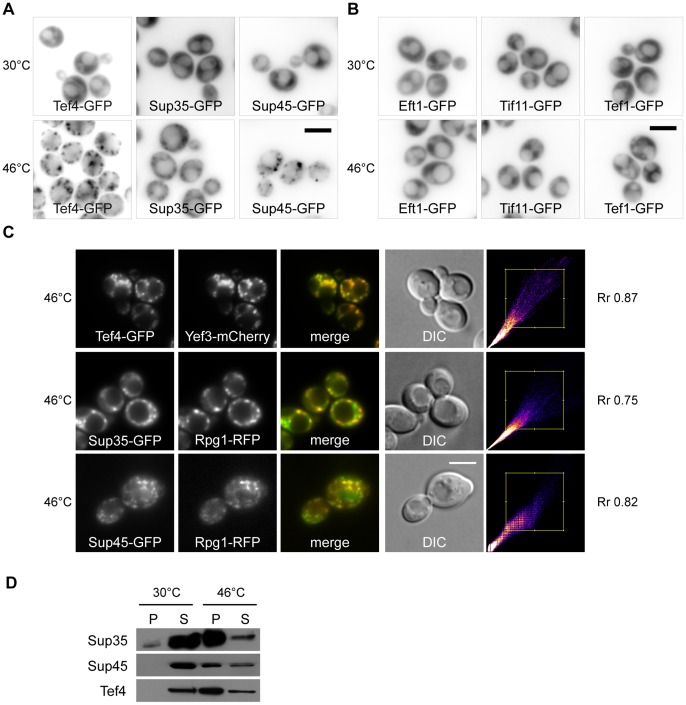
Factors eEF1Bγ2 (Tef4p), eRF1 (Sup45p) and eRF3 (Sup35p) are novel components of stress granules. (A) The distribution of Tef4-GFP (CRY554 strain), Sup35-GFP (CRY412 strain) and Sup45-GFP (CRY1552 strain) proteins was analyzed in control (30°C) and heat-shocked (46°C) cells. All of these fusion proteins accumulated in discrete cytoplasmic foci in heat-shocked cells. (B) Subcellular localization of Eft1-GFP (eEF2) (CRY552 strain), Tif11-GFP (eIF1A) (CRY521 strain) and Tef1-GFP (eEF1A) (CRY1764 strain) fusion proteins upon control and heat shock conditions. The distribution of Eft1-GFP, Tif11-GFP and Tef1-GFP remained diffusely cytosolic even upon the stress condition. (C) Tef4-GFP fusion protein co-localized with accumulations of the Yef3-mCherry fusion protein (CRY1315 strain). Similarly, Sup35-GFP and Sup45-GFP co-localized with the stress granule marker protein Rpg1-RFP in cells heat-shocked at 46°C for 10 minutes (strains CRY1364; CRY1627 strains). High values of the Pearsońs correlation coefficient (R_r_) confirmed the high degree of this co-localization. Scale bar 4 µm. (D) Pellet and supernatant fractions from the appropriate cell lysates (strains CRY554; CRY412; CRY1552 strains) were analyzed by SDS-PAGE and Western blotting. Samples were prepared as described elsewhere [2]. All tested fusion proteins were clearly accumulated in the pellet fraction of cells heat-shocked at 46°C for 10 minutes.

Since we found that heat-induced SGs contain the translation elongation factor eEF3, we wanted to know whether other translation factors are also present. Using the strains from the GFP collection [18], we analyzed the distribution of some other translation factors and found that the elongation factor eEF1B*γ*2 (Tef4p) together with the termination factors eRF3 (Sup35p) and eRF1 (Sup45p) also accumulate in distinct cytoplasmic foci under heat shock at 46°C (Fig. 3A). Other microscopic analyses using the particular strains from the GFP collection [18] revealed that other translation factors, elongation factor eEF1A (Tef1p), elongation factor eEF2 (Eft1p) and initiation factor eIF1A (Tif11p), remained uniformly cytosolic when the robust heat shock was applied (Fig. 3B).

To co-localize Tef4, Sup45 and Sup35 heat-induced foci with SGs, we prepared a strain co-expressing Tef4-GFP and Yef3-mCherry fusion proteins and strains co-expressing Sup45-GFP or Sup35-GFP fusion proteins together with the stress granule marker Rpg1-RFP. As shown in Fig. 3C, there is a near-perfect overlap of accumulated Tef4-GFP and Yef3-mCherry fusion proteins upon robust heat shock. Similarly, Sup35-GFP or Sup45-GFP significantly overlapped with the accumulated Rpg1-RFP in heat-shocked cells (Fig. 3C). In addition, similar results were obtained in the strains co-expressing either Sup45-GFP and Dcp2-mCherry or Sup45-GFP and Ngr1-mCherry fusions (Fig. S1).

Changes in the subcellular distribution of Tef4-GFP, Sup35-GFP and Sup45-GFP fusion proteins upon robust heat shock were confirmed by SDS-PAGE and the Western blot analysis of samples prepared by differential centrifugation (Fig. 3D). In agreement with the results of microscopic analyses, the tested proteins were found to be enriched in the pellet fraction of heat-shocked cells. We thus established eEF1γ2 (Tef4p), eRF1 (Sup45p) and eRF3 (Sup35p) factors to be novel constituents of SGs induced by robust heat shock in *S. cerevisiae*.

### The N-terminal Prion-like Domain of Sup35 Protein is not Involved in SGs Assembly

**Figure 4:**
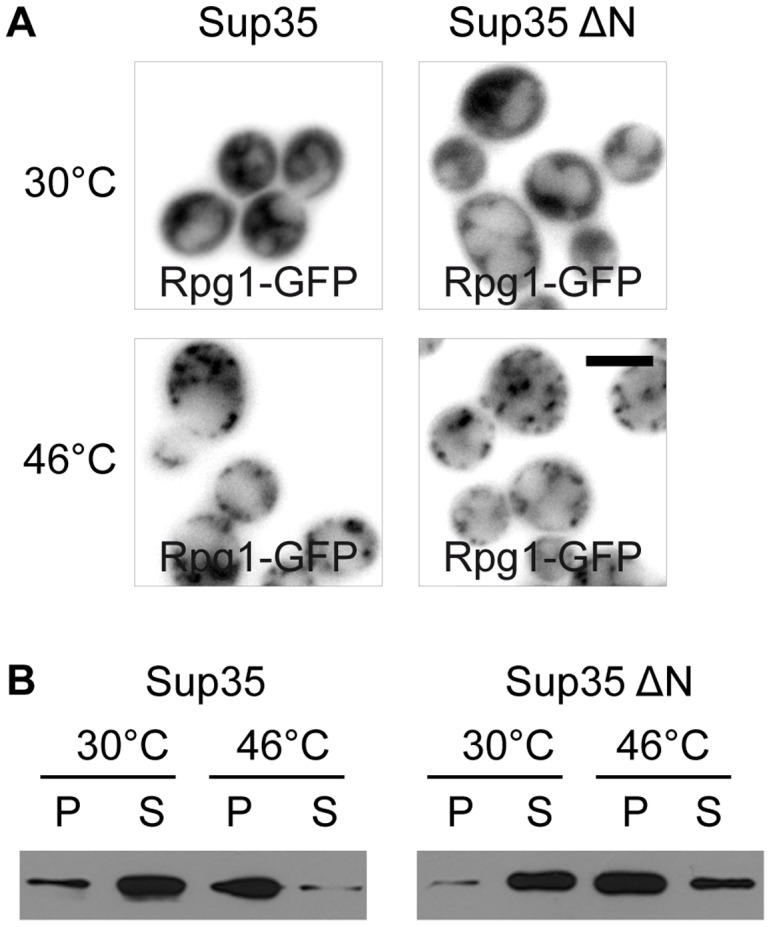
Full length Sup35 protein is not necessary for SGs assembly and its localization into SGs. (A) The distribution of Rpg1-GFP fusion protein under control (30°C) and heat shock (46°C) conditions in either the wild-type strain (Sup35p; CRY1011 strain) or the strain expressing only the N-terminal truncated form of Sup35 protein (Sup35ΔN; CRY1007 strain). SGs (Rpg1-GFP) were formed even in the mutant strain suggesting that the N-terminal part of Sup35 protein is dispensable for the SGs formation. Scale bar 4 µm. (B) Pellet and supernatant fractions from the wild-type (CRY998 strain) and the Sup35 N-terminal truncated mutant strains (CRY1001 strain) were analyzed by SDS-PAGE and Western blotting using the anti-Sup35 antibody. Samples were prepared as described elsewhere [2]. The N-terminal truncated form of Sup35 protein was enriched in the pellet fraction of heat-shocked cells, as well as the wild-type form of Sup35 protein, suggesting that only Sup35 C-terminal part is sufficient to localize this protein to the heat-induced stress granules.

As described above, we identified the translation termination factor eRF3 (Sup35p) as a constituent of SGs induced by robust heat shock at 46°C. Interestingly, this protein belongs to known yeast prion proteins and its N-terminal part is indispensable for [PSI+] prion formation and maintenance [38], [39]. Since some proteins with prion-like domains have been referred to as affecting development of SGs in mammalian cells [40], we wanted to understand whether the prion-induction domain of Sup35 protein affects accumulation of Rpg1/eIF3a, the SGs marker protein. We constructed the strain expressing only the essential C-terminal part of Sup35 protein together with Rpg1-GFP. As a control, we used the strain of the same genetic background co-expressing the wild-type form of Sup35p and the Rpg1-GFP fusion protein. We observed Rgp1-GFP accumulated in SGs of both, wild-type and the mutant cells heat-shocked at 46°C for 10 minutes (Fig. 4A) suggesting that assembly of heat-induced SGs does not require the N-terminal part of Sup35 protein. In addition, using differential centrifugation followed by SDS-PAGE and Western blotting we observed that even in the absence of the Sup35 N-terminal domain the truncated protein was still accumulated in SGs of the cells heat-shocked at 46°C (Fig. 4B). This indicates that the full length Sup35 protein can be accumulated in SGs in a non-prion form. We conclude that the prion-induction domain of Sup35 protein is not necessary for SG assembly and the C-terminal part of this protein is sufficient to recruit the whole Sup35 protein to SGs.

### Translation Elongation Factor eEF3 (Yef3p) Accumulates in RNP Complexes Even at 42°C

**Figure 5:**
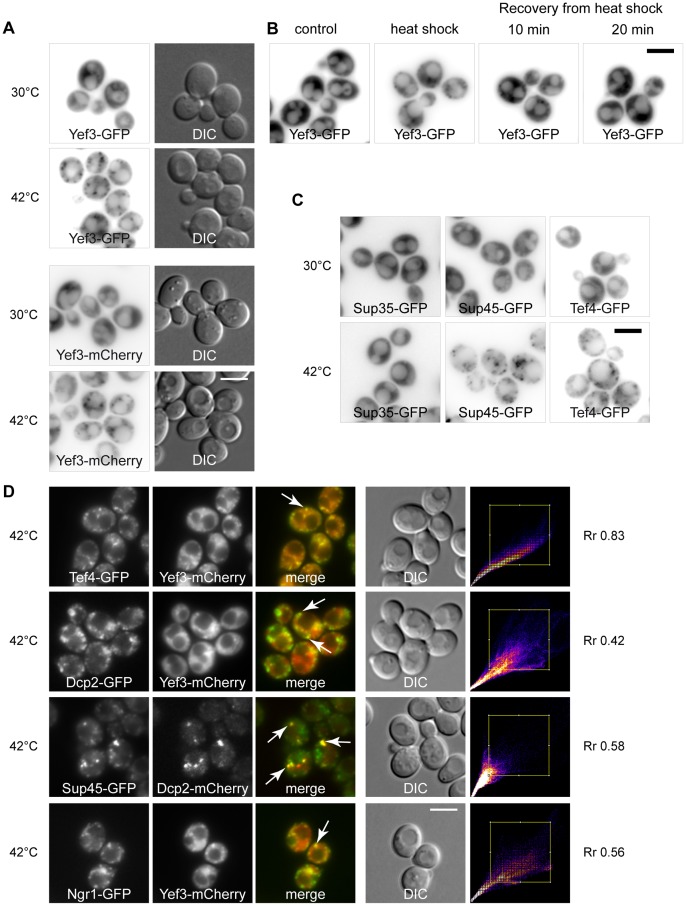
Translation elongation and termination factors accumulate in the same foci at 42 °**C.** (A) The distribution of Yef3-GFP or Yef3-mCherry fusion protein was analyzed in wild-type cells (CRY510 strain: CRY1339 strain) heat-shocked at 42°C for 10 minutes. Whereas the Yef3-GFP and Yef3-mCherry fusion protein were uniformly cytosolic at 30°C, they formed discrete cytoplasmic foci in the cells heat-shocked at 42°C for 10 minutes. (B) Distribution of the Yef3-GFP protein (CRY510 strain) was analyzed in control, heat-shocked (at 42°C) and recovering cells. Yef3-GFP accumulations were dissolved within 20 minutes of the cell recovery from the stress. (C) The distribution of Sup35-GFP (CRY412 strain), Sup45-GFP (CRY1552 strain) and Tef4-GFP (CRY554 strain) was analyzed under control conditions at 30°C and under heat shock at 42°C. Whereas Sup35-GFP protein remained uniformly cytosolic even upon the heat shock at 42°C, Sup45-GFP and Tef4-GFP proteins accumulated into discrete cytoplasmic foci (D) Yef3-mCherry fusion protein was co-localized either with Tef4-GFP (CRY1315 strain), Dcp2-GFP (CRY1559 strain) or Ngr1-GFP (CRY1287 strain) fusion proteins, as well as Sup45-GFP fusion protein was co-localized with Dcp2-mCherry fusion protein (CRY1636 strain), in cells heat-shocked at 42°C.

Despite the fact that eEF3 factor (Yef3p) was not significantly enriched in the pellet fraction of the cells heat-shocked at 42°C (see Fig. 1A), we also analyzed the distribution of this protein by fluorescence microscopy under these stress conditions. Surprisingly, Yef3-GFP or Yef3-mCherry accumulated in cytoplasmic foci even upon heat shock at 42°C (Fig. 5A). It should be noted that Yef3-mCherry fusion protein forms fewer granules in comparison to Yef3-GFP protein under heat shock at 42°C. The accumulations of eEF3 (Yef3-GFP) after heat shock at 42°C for 10 minutes were transient and dissolved after releasing cells from the stress conditions (Fig. 5B).

We also analyzed distribution of other newly identified components of SGs, e.g. Tef4, Sup45 and Sup35 proteins, upon the heat shock at 42°C. Despite we did not see accumulation of Sup35-GFP, we were able to observe formation of visible assemblies of Tef4-GFP and Sup45-GFP under these stress conditions (Fig. 5C).

Subsequent microscopic analyses of strains co-expressing Tef4-GFP with Yef3-mCherry, Dcp2-GFP with Yef3-mCherry, Sup45-GFP with Dcp2-mCherry and Ngr1-GFP with Yef3-mCherry from the chromosomal sites confirmed that all these proteins co-localized in distinct foci in cells heat-shocked at 42°C for 10 minutes (Fig. 5D). Similar results were obtained for the strains co-expressing Ngr1-GFP with Dcp2-mCherry and Pub1-GFP with Dcp2-RFP fusion proteins (data not shown).

**Figure 6:**
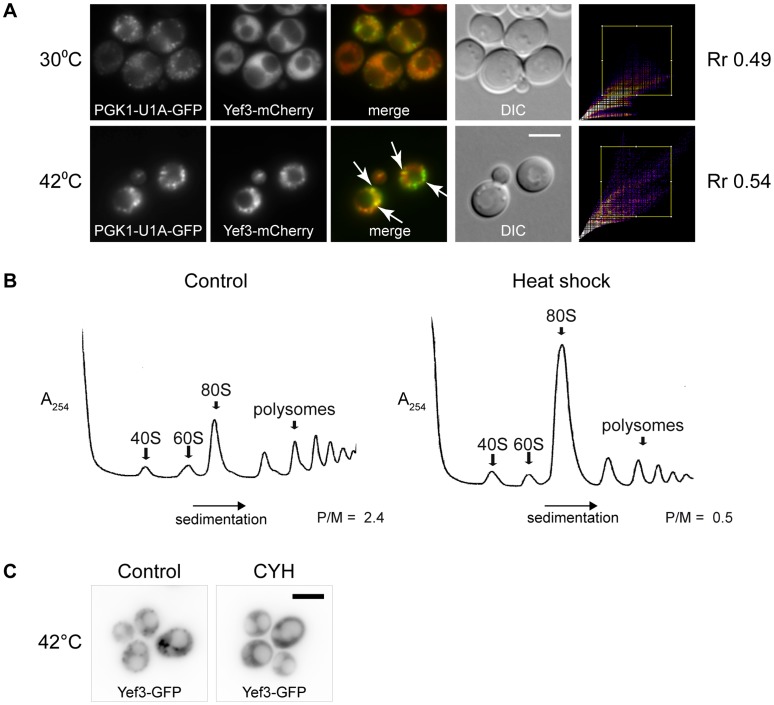
Heat shock at 42°C alters translation and triggers accumulation of mRNA in Yef3 foci. (A) Distribution of *PGK1* mRNA (*PGK1* mRNA/U1A-GFP reporter system) at 30°C or upon heat shock at 42°C was analyzed in cells expressing Yef3-mCherry fusion protein (CRY1618 strain). Fine granules of *PGK1* mRNA (U1A-GFP) were present at 30°C in a majority of cells. The heat shock at 42°C induced an accumulation of some of these granules into larger clusters and formations of Yef3 foci. Arrows point to obviously overlapping signals of both fusions. Scale bar 4 µm. (B) Polysome profiles of wild-type strain (BY4741) at permissive temperature and after heat shock at 42°C for 10 minutes. Translation profile was altered in heat-shocked cells. P/M stands for polysome/monosome ratio. (C) Distribution of Yef3-GFP (CRY510 strain) was analyzed in cells heat-shocked at 42°C for 10 minutes with or without preincubation in the presence of cycloheximide (CYH). When this drug was added before the heat shock, no accumulations of Yef3-GFP protein were formed. Scale bar 4 µm.

To determine whether the Yef3-containing foci formed at 42°C comprise mRNA, we used the *PGK1* mRNA/U1A-GFP detection reporter system [9]. Initially, we observed that fine granules of *PGK1* mRNA (U1A-GFP) were present even under control temperature in a majority of cells. The heat shock at 42°C induced accumulation of some of these granules into larger clusters. We observed co-localization of the *PGK1* mRNA (U1A-GFP) clusters with accumulations of Yef3-mCherry fusion protein under these heat shock conditions (Fig. 6A). We used the “Co-localization Finder” plugin (ImageJ) to express statistically the overlaps. The value of the Pearson’s correlation coefficient (R_r_) for both cultures about 0.5 indicates co-localization, but also means that there is always a portion of Yef3 translation elongation factor which is not directly associated with the mRNA-U1A-GFP and vice versa. We supposed that the translation polysome profiles of the heat-shocked cells would be altered at 42°C. Indeed, as shown in Fig. 6B, the polysomal fraction was smaller and the 80S peak enlarged for cells heat-shocked at 42°C comparing the polysome profile of non-stressed cells. This suggested that mRNA molecules leave the active translation cycle and accumulate in cytoplasmic Yef3-containing RNP complexes. To support these results, we performed experiments in which mRNA was trapped in polysomes after cycloheximide (CYH) pretreatment. As expected, Yef3-containing accumulations were not assembled in cells pretreated with CYH and heat-shocked at 42°C for 10 minutes (Fig. 6C). This confirms that free mRNA outside the ribosome (polysome) is a prerequisite for the formation of RNP accumulations upon heat shock at 42°C.

We published earlier [2] that formation of heat-induced SGs and translation arrest accompanying robust heat shock are independent of Gcn2 kinase. In accordance with these findings, we observed that formation of Yef3-containing foci at 42°C and the alteration of polysome profile seen at 42°C are also Gcn2 independent (Fig. S2).

### Accumulations of RNP Complexes Formed at 42°C Determine Sites for SGs Assembled at 46°C

**Figure 7:**
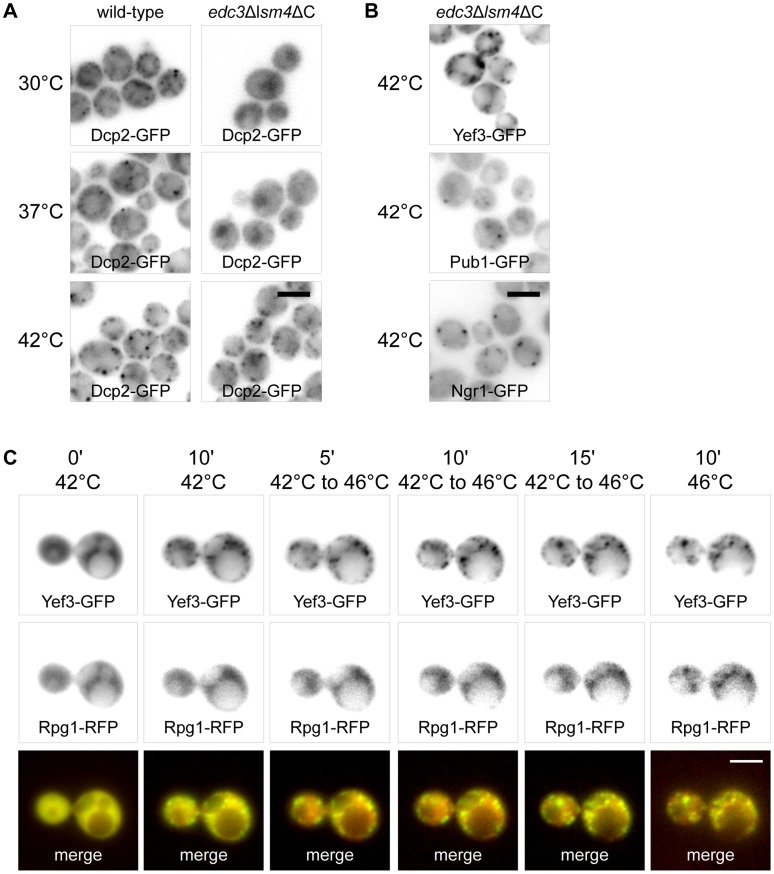
RNP accumulations formed at 42°C serve as “seeds” for genuine SGs. (A) Distribution of Dcp2-GFP was analyzed in wild-type (CRY564 strain) and *edc3*Δ*lsm4*ΔC mutant (CRY977 strain) cells after incubation at 30°C, 37°C and 42°C for 10 minutes. These wild-type cells exhibited increasing number of enlarged accumulations of the Dcp2-GFP fusion protein corresponding to raising temperatures of the heat shock. In contrast, *edc3*Δ*lsm4*ΔC mutant cells did not exhibit any cytoplasmic accumulations of the Dcp2-GFP fusion protein upon incubation at 30°C or 37°C. However, these mutant cells displayed accumulations of Dcp2-GFP upon heat shock at 42°C for 10 minutes. (B) The distribution of fusion proteins Pub1-GFP, Ngr1-GFP and Yef3-GFP was analyzed in *edc3*Δ*lsm4*ΔC mutant cells (CRY1035, CRY1041 and CRY1146 strains) either at 30°C or upon heat shock at 42°C for 10 minutes. All of these proteins accumulated in the mutant cells at 42°C, thus independently of P-body scaffolding proteins Edc3 and Lsm4. (C) Time lapse experiment using the cells co-expressing Yef3-GFP and Rpg1-RFP fusion proteins (CRY649 strain) showing the formation of Yef3-GFP foci after 10 minutes at 42°C, accumulation of Rpg1-RFP protein during the temperature rise from 42°C to 46°C and after 10 minutes at 46°C. Yef3-GFP foci were first formed at 42°C, whereas the localization of the Rpg1-RFP protein was still uniformly cytoplasmic at this temperature. During heating from 42°C to 46°C and upon cultivation at 46°C, the Rpg1-RFP protein only accumulated at the preformed Yef3-GFP foci. Scale bar 4 µm.

To analyze the RNP accumulations formed at 42°C in detail, we used the *edc3*Δ*lsm4*ΔC mutant, which is unable to form visible P-bodies [8]. We prepared appropriate *edc3*Δ*lsm4*ΔC mutant strains expressing GFP fusion variants of some proteins found to accumulate in joint assemblies under heat shock at 42°C (see above), e.g. Yef3p, Dcp2p, Ngr1p and Pub1p. We found that in contrast to wild-type cells, the *edc3*Δ*lsm4*ΔC mutant did not exhibit any accumulations of the Dcp2-GFP fusion protein under control conditions at 30°C or upon heat shock at 37°C (Fig. 7A). These cells only displayed the Dcp2-GFP signal in the nuclear region. However, the mutant cells displayed distinct accumulations of Dcp2-GFP under heat shock at 42°C. We conclude that these Dcp2-GFP accumulations at 42°C do not depend on P-bodies scaffolding proteins Edc3 and Lsm4. Interestingly, we found that also accumulations of other proteins Ngr1-GFP, Pub1-GFP and Yef3-GFP at 42°C was independent on Edc3 and Lsm4 proteins (Fig. 7B). The distribution pattern of these protein accumulations in the mutant was similar to that of wild-type cells. We conclude that Dcp2-containing accumulations at 42°C, similarly to other components of SGs, do not require P-bodies scaffolding proteins.

The similarity of RNP accumulations formed at 42°C to SGs formed at 46°C indicated that they are structurally related. We performed a time-lapse experiment using the strain co-expressing Yef3-GFP with the SGs marker protein Rpg1-RFP (Fig. 7C). The cells were pre-heated to 42°C and then continuously heated up to 46°C. The Yef3-GFP fusion protein was accumulated during the initial cultivation at 42°C and also later on. In contrast, the Rpg1-RFP fusion protein began to accumulate around Yef3-GFP foci only after raising the temperature above 42°C, with most focused accumulations being observed at 46°C. We conclude that the RNP accumulations formed at 42°C serve as “seeds” for genuine SGs assembled upon robust heat shock at 46°C.

### Translation Initiation Factor eIF2α Subunit is Recruited to Heat-induced SGs Upon a Stress Relief

**Figure 8:**
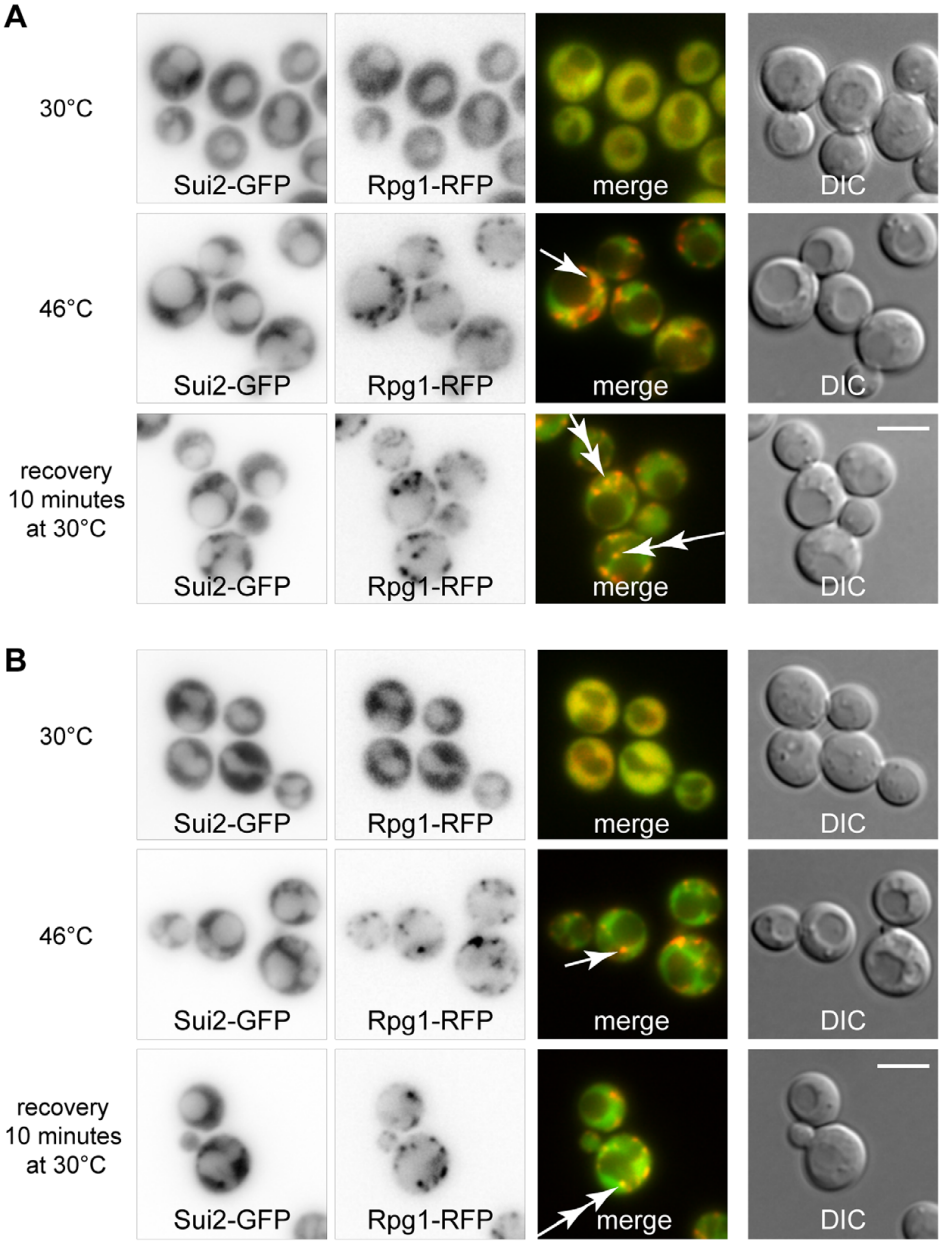
Sui2p associates with dissolving SGs in cells recovering from the heat shock. (A) The localization of fusion proteins Sui2-GFP and Rpg1-RFP was analyzed in cells (CRY1332 strain) cultivated at 30°C or heat-shocked at 46°C for 10 minutes or cultivated after heat shock for additional 10 minutes. Sui2-RFP protein was not accumulated in stress granules (Rpg1-GFP) of heat-shocked cells (single arrow). Sui2 protein began to accumulate with stress granules during the cell recovery phase after cultivation of the heat-shocked cells at 30°C for additional 10 minutes (double arrows). (B) The same situation as in (A), but in the *gcn2*Δ strain (CRY1691 strain). Scale bar 4 µm.

In contrast to P-bodies, the composition of SGs suggests that SGs might be sites where translation initiates during the cell recovery from a stress. In our previous work [2], we described that α subunit of the translation initiation factor eIF2 (Sui2p) is not a constituent of the robust heat shock-induced SGs in *S. cerevisiae*. Here we analyzed distribution of the Sui2 protein in cells recovering from the heat shock using a strain co-expressing Sui2-GFP fusion protein with the stress granule marker Rpg1-RFP. In contrast to the cells heat-shocked at 46°C, where distribution of Sui2-GFP was uniformly cytosolic, the cells recovering from the heat shock displayed accumulations of Sui2-GFP overlapping with dissolving SGs (Rpg1-RFP) (Fig. 8A).

To analyze whether accumulation of Sui2-GFP on SGs during the recovery from the stress is dependent on a phosphorylation status of Sui2p, we used a new strain carrying Sui2-GFP and Rpg1-RFP proteins together with the *gcn2* deletion. We found that Sui2-GFP accumulated on dissolving SGs even in the absence of the Gcn2 kinase (Fig. 8B). It means that Sui2p accumulated on SGs is present also in its non-phosphorylated form, thus translation competent. Our data thus support the hypothesis that SGs serve as sites to initiate translation after a stress relief.

## Discussion

It is widely accepted that stress granules (SGs) are composed of stalled translation preinitiation complexes (48S) containing mRNA, small ribosomal subunits and some translation initiation factors [15], [41]. Here we provide evidence that the heat-induced SGs formed at 46°C in *S. cerevisiae* also contain certain translation elongation and translation termination factors. Some of them, elongation factors eEF3 and eEF1Bγ2 and the termination factor eRF1 accumulate already at 42°C on Dcp2 foci independently of P-bodies scaffolding proteins. We also showed, on the example of eEF3 factor, that an accumulation of the protein at 42°C is independent on the Gcn2 kinase activity. However, these foci still determine sites for assembly of SGs upon heat shock at 46°C. Our data suggest that assembly of these SGs might be controlled by translation elongation and termination factors released from ongoing translation. Furthermore, recruitment of the key translation initiation factor eIF2α (Sui2p) to dissolving SGs points to the recovery of translation at these sites after stress relief.

Although SGs are referred to as stalled translation preinitiation complexes, it is generally accepted that the composition of SGs varies depending on organisms and cell types, as well as on intensity and type of the stress [42]. For example, SGs induced by a high concentration of ethanol in *S. cerevisiae* contain only the eIF3c/Nip1 subunit of the eIF3 complex [3] and SGs induced by a prolonged glucose deprivation do not harbor the eIF3 complex at all [1]. With respect to the intensity of the stress, treatment with a low concentration of NaN_3_ does not affect the distribution of eIF3a (Rpg1p/Tif32p) [2] but at higher concentration this drug induces eIF3a accumulation in SGs [17]. We show here that SGs induced by the robust heat shock in *S. cerevisiae* contain translation elongation factors eEF3 (Yef3p) and eEF1Bγ2 (Tef4p) together with translation termination factors eRF1 (Sup45p) and eRF3 (Sup35p). These factors have never been observed in SGs of any other eukaryotic cell. However, the termination factors have been found to accumulate in P-bodies [1], [43]. Those authors have concluded that presence of translation termination factors in P-bodies is coupled to the P-bodies assembly. A similar role could be suggested for presence of these factors in heat-induced SGs.

The proteins with self-aggregation (prion-like) domain, like TIA-1 or TIAR in mammalian cells, have been described to influence dynamics of SGs [40]. A newly identified component of the heat-induced SGs in *S.cerevisiae*, Sup35p, possesses a prion-like domain at the N-terminus. Sup35p can thus convert into the prion form, known as [PSI+]. The N-terminal part of the protein is indispensable for the prion formation and maintenance [38], [39]. Similarly to a situation in mammalian cells [40] we found that rather a non-prion part of Sup35p is responsible for accumulation of the protein in SGs. However, observations that SGs are formed even in the absence of the N-terminal prion-like domain of Sup35p indicate that unlike in mammals, the assembly of heat-induced SGs in *S. cerevisiae* is not driven by these “prion” structural elements. This hypothesis is also supported by our earlier findings that heat-induced SGs are formed even in the absence of yeast orthologs of mammalian TIA-1 and TIAR proteins, Ngr1 and Pub1 proteins in *S. cerevisiae* [2].

Translation termination factors eRF1 (Sup45p) and eRF3 (Sup35p) are responsible for effective termination of translation [44]. In addition, they seem to be required for an effective function of the fungal-specific elongation factor eEF3 (Yef3p) [45] in recycling of the translation posttermination complexes after the release of newly synthetized peptide chains [46], [47]. In this respect, identification of elongation and termination factors in heat-induced SGs may indicate that these SGs are composed of translation posttermination complexes stalled before the ribosome recycling step. However, we did not observe any accumulation of several essential proteins of the 60S ribosomal subunits under robust heat shock [2] and Grousl et al. (unpublished data). In addition, all the published information on recycling of the translation posttermination complexes comes from in vitro experiments only. Therefore, it is currently unclear, how recycling is catalyzed in vivo and the reasons for presence of the translation elongation and the termination factors in robust heat shock-induced SGs remain to be elucidated.

Whereas different roles for SGs and P-bodies in cell survival upon heat stress conditions could be suggested, both accumulations are always closely spatially and functionally intertwined. In *S. cerevisiae* cells, P-bodies promote formation of SGs [1], [2]. The assembly of P-bodies is connected with changes in expression profiles and adaptation to changed environmental conditions, when the translation of certain transcripts is inhibited and, on the other hand, the translation of new transcripts is induced [11]. They are present in cells even under non-stress conditions and they enlarge under various stresses, such as a heat shock at 39°C [21] and 42°C [48]. However, one of the major components of P-bodies, Dcp2p that is engaged in mRNA decapping [5], [9], [10], is also a component of the robust heat shock-induced SGs, which may form independently of the P-bodies scaffolding proteins Edc3 and Lsm4 [2]. On the contrary to glucose-deprived cells [1], [8] and cells heat-shocked at 37°C, we show here that Dcp2 foci formed in cells heat-shocked at 42°C do not depend on these scaffolds. These Dcp2 accumulations do not contain translation initiation factors and still serve as sites for assembly of SGs upon continuous and more robust heat stress. They might be considered as “premature SGs”, as well as Dcp2-containing structures related to P-bodies.

The stress-induced phosphorylation of translation initiation factor eIF2α is the best characterized mechanism of stress granule assembly [14], [15]. However, there are other ways, how to induce SGs or influence their dynamics. Apart influencing other translation initiation factors, they concern the metabolism of polyamines or hexosamines and the stress-induced tRNA derivates [29], [31], [49], [50]. Moreover, since eIF2α-phosphorylation independent mechanisms of SGs assembly prevail in lower eukaryotes, it seems that these are evolutionary older. We show here that accumulations of translation elongation factor eEF3 (Yef3p) on Dcp2 foci at 42°C precede assembly of eIF3-containing SGs in cells heat-shocked at 46°C. This suggests that the translation elongation phase is affected first in the stressed cells. It is conceivable that there might be a shortage of the eEF3 factor due to its sequestration into cytoplasmic foci at 42°C. This may cause an alteration of kinetics of the translation elongation, which results in a deceleration of translation initiation. Such regulation of translation at the elongation step has also been proposed as a possible function of the Stm1 protein, which is able to stall ribosomes after the 80S complex formation *in vitro* and to promote decapping of a subset of mRNA [51]. Moreover, Stm1p has been shown to regulate interaction of eEF3 factor with ribosomes and to play a complementary role to eEF3 in translation under nutrient stress conditions [52]. Interestingly, we did not observe an accumulation of Stm1-GFP fusion protein neither upon heat shock at 42°C nor under heat shock at 46°C (Figure S3). Additionally, we did not see any effect of the *stm1*Δ on assembly of SGs in cells heat-shocked at 46°C (Figure S4). Therefore, the roles of Stm1 protein and eEF3 factor in the Gcn2-independent signaling and translation repression resulting in SGs assembly in heat-shocked cells remain elusive.

Meanwhile assembly of P-bodies is generally connected with reprogramming of cells to new growth conditions, SGs are formed in response to severe stresses when the translation of housekeeping genes is completely shut down. Although SGs are thought to be sites where mRNA molecules are sorted, selected, and together with translation factors, sheltered from the effects of a stress [15], [53], the fate of SGs components after a stress relief is mainly unknown [42]. However, it is conceivable that at least some SGs protein components may also return back to the active translation. In this respect, our observation of accumulation of the key translation initiation factor eIF2α (Sui2p) on dissolving SGs during cell recovery from the heat stress suggests that SGs may help cells to effectively recover after a stress relief. To recover, a fast restart of translation is facilitated by increasing a local concentration of translation initiation and elongation components in SGs, where only the key regulator, eIF2α factor (Sui2p), is missing and recruited after a stress relief only. There is a supporting evidence from mammalian cells where a phospho-variant of the eIF2α factor subunit was found to be recruited to disassembling SGs and considered as important for SGs disassembly [15]. On the contrary, we found that the eIF2α factor (Sui2p) accumulation on SGs does not depend on the phosphorylation status of this factor. It implies that the eIF2α factor is recruited to dissolving SGs also in its unphosphorylated state, thus translation competent. Taken together, it reinforces the hypothesis that SGs serve as sites where translation is effectively initiated at the time of a stress relief.

We showed here that a portion of the key translation initiation factor eIF2α (Sui2p) is recruited to dissolving SGs, but some of the Sui2-GFP foci did not co-localize with SGs markers in these cells. We suggest that these particular Sui2-GFP foci may represent the eIF2B bodies [54]. In accordance with our assumption that translation is restored on dissolving SGs, the eIF2B bodies should also be formed under recovery from the stress. The eIF2B bodies most probably serve as sites, where guanine nucleotide exchange of the eIF2α factor takes place and the eIF2α-GDP form is converted to the translation competent eIF2α-GTP form. The eIF2B bodies would then help to regenerate efficiently the translation competent form of the eIF2α factor as suggested previously [55]. The eIF2α-GTP form would then be recruited to sites on dissolving SGs.

Altogether, our data support the current view that the composition of stress granules depends on the type and the intensity of the applied stress. We confirmed that formation of yeast heat shock-induced SGs is not dependent on the translation initiation arrest caused by phosphorylation of eIF2α and we propose that translation machinery in heat shocked-cells seems to be primarily modulated at the level of translation elongation since also some translation elongation and termination factors accumulate within SGs. Our data further indicate that SGs reflect the sites where translation initiates after a stress relief. We also show that RNP accumulations formed upon heat shock at 42°C and containing translation elongation and termination factors may develop into genuine SGs upon robust heat shock at 46°C. Although we confirmed that all these accumulations depend on mRNA released from translation, links between heat-induced repression of translation and SGs assembly still remain to be elucidated.

## Supporting Information

Figure S1 **Factor eRF1 (Sup45p) co-localizes with Dcp2 and Ngr1 proteins in stress granules.** (A) The subcellular distribution of Sup45-GFP and Dcp2-mCherry (CRY1636 strain) or (B) Sup45-GFP and Ngr1-mCherry (CRY1638) fusion proteins under heat shock at 46°C. Both pairs of proteins co-localize within stress granules. Values of the Pearsońs correlation coefficient (R_r_) over 0.5 confirmed the co-localization. Scale bar 4 µm.
(TIF)

Figure S2 **An accumulation of eEF3 factor and an alteration of polysome profile at 42°C are** ***gcn2*** **Δ independent.** (A) The distribution of Yef3-GFP protein in *gcn2*Δ strain (CRY1336 strain) at 30°C and under heat shock at 42°C. The protein accumulates in the same extend as the wild-type strain (compare with Figure 5) upon the heat shock. (B) Polysome profiles of *gcn2*Δ strain (CRY309 strain) at permissive temperature and after heat shock at 42°C for 10 minutes. Translation profile was altered in heat-shocked cells. P/M stands for polysome/monosome ratio.
(TIF)

Figure S3 **Subcellular distribution of Stm1-GFP fusion protein is not altered upon heat shock at 42°C and 46°C.** The distribution of Stm1-GFP fusion protein (CRY1516 strain) remains diffusely cytosolic under all tested conditions. Scale bar 4 µm.
(TIF)

Figure S4 **Deletion of the** ***STM1*** **gene does not affect assembly of heat-induced SGs.** (A) The distribution of Pab1-GFP and Rpg1-RFP proteins in wild-type cells (CRY528 strain) at 30°C and upon heat shock at 46°C for 10 minutes. Both proteins are accumulated in stress granules. (B) The *stm1*Δ mutant (CRY1760 strain) displayed a similar pattern of stress granules as the wild-type cells. Scale bar 4 µm.
(TIF)

## References

[1] Buchan JR, Muhlrad D, Parker R (2008) P bodies promote stress granule assembly in Saccharomyces cerevisiae. J Cell Biol 183: 441–455. 18981231 10.1083/jcb.200807043 PMC2575786

[2] Grousl T, Ivanov P, Frydlova I, Vasicova P, Janda F, et al (2009) Robust heat shock induces eIF2alpha-phosphorylation-independent assembly of stress granules containing eIF3 and 40S ribosomal subunits in budding yeast, Saccharomyces cerevisiae. J Cell Sci 122: 2078–2088. 19470581 10.1242/jcs.045104

[3] Kato K, Yamamoto Y, Izawa S (2011) Severe ethanol stress induces assembly of stress granules in Saccharomyces cerevisiae. Yeast 28: 339–347. 21341306 10.1002/yea.1842

[4] Nilsson D, Sunnerhagen P (2011) Cellular stress induces cytoplasmic RNA granules in fission yeast. RNA 17: 120–133. 21098141 10.1261/rna.2268111 PMC3004053

[5] Sheth U, Parker R (2003) Decapping and decay of messenger RNA occur in cytoplasmic processing bodies. Science 300: 805–808. 12730603 10.1126/science.1082320 PMC1876714

[6] Hofmann S, Cherkasova V, Bankhead P, Bukau B, Stoecklin G (2012) Translation suppression promotes stress granule formation and cell survival in response to cold shock. Mol Biol Cell 23: 3786–3800. 22875991 10.1091/mbc.E12-04-0296 PMC3459856

[7] Eulalio A, Behm-Ansmant I, Izaurralde E (2007) P bodies: at the crossroads of post-transcriptional pathways. Nat Rev Mol Cell Biol 8: 9–22. 17183357 10.1038/nrm2080

[8] Decker CJ, Teixeira D, Parker R (2007) Edc3p and a glutamine/asparagine-rich domain of Lsm4p function in processing body assembly in Saccharomyces cerevisiae. J Cell Biol 179: 437–449. 17984320 10.1083/jcb.200704147 PMC2064791

[9] Brengues M, Teixeira D, Parker R (2005) Movement of eukaryotic mRNAs between polysomes and cytoplasmic processing bodies. Science 310: 486–489. 16141371 10.1126/science.1115791 PMC1863069

[10] Balagopal V, Parker R (2009) Polysomes, P bodies and stress granules: states and fates of eukaryotic mRNAs. Curr Opin Cell Biol 21: 403–408. 19394210 10.1016/j.ceb.2009.03.005 PMC2740377

[11] Arribere JA, Doudna JA, Gilbert WV (2011) Reconsidering movement of eukaryotic mRNAs between polysomes and P bodies. Mol Cell 44: 745–758. 22152478 10.1016/j.molcel.2011.09.019 PMC3240842

[12] Parker R (2012) RNA Degradation in Saccharomyces cerevisae. Genetics 191: 671–702. 22785621 10.1534/genetics.111.137265 PMC3389967

[13] Balagopal V, Fluch L, Nissan T (2012) Ways and means of eukaryotic mRNA decay. Biochim Biophys Acta. 10.1016/j.bbagrm.2012.01.001 22266130

[14] Anderson P, Kedersha N (2008) Stress granules: the Tao of RNA triage. Trends Biochem Sci 33: 141–150. 18291657 10.1016/j.tibs.2007.12.003

[15] Anderson P, Kedersha N (2002) Stressful initiations. J Cell Sci 115: 3227–3234. 12140254 10.1242/jcs.115.16.3227

[16] Takahara T, Maeda T (2012) Transient Sequestration of TORC1 into Stress Granules during Heat Stress. Mol Cell 47: 242–252. 22727621 10.1016/j.molcel.2012.05.019

[17] Buchan JR, Yoon JH, Parker R (2011) Stress-specific composition, assembly and kinetics of stress granules in Saccharomyces cerevisiae. J Cell Sci 124: 228–239. 21172806 10.1242/jcs.078444 PMC3010191

[18] Huh WK, Falvo JV, Gerke LC, Carroll AS, Howson RW, et al (2003) Global analysis of protein localization in budding yeast. Nature 425: 686–691. 14562095 10.1038/nature02026

[19] Dunand-Sauthier I, Walker C, Wilkinson C, Gordon C, Crane R, et al (2002) Sum1, a component of the fission yeast eIF3 translation initiation complex, is rapidly relocalized during environmental stress and interacts with components of the 26S proteasome. Mol Biol Cell 13: 1626–1640. 12006658 10.1091/mbc.01-06-0301 PMC111132

[20] Hoyle NP, Castelli LM, Campbell SG, Holmes LE, Ashe MP (2007) Stress-dependent relocalization of translationally primed mRNPs to cytoplasmic granules that are kinetically and spatially distinct from P-bodies. J Cell Biol 179: 65–74. 17908917 10.1083/jcb.200707010 PMC2064737

[21] Cowart LA, Gandy JL, Tholanikunnel B, Hannun YA (2010) Sphingolipids mediate formation of mRNA processing bodies during the heat-stress response of Saccharomyces cerevisiae. Biochem J 431: 31–38. 20629639 10.1042/BJ20100307 PMC3804835

[22] Proud CG (2005) Semin Cell Dev Biol. 16: 3–12. 10.1016/j.semcdb.2004.11.004 15659334

[23] Hinnebusch AG (2005) eIF2alpha kinases provide a new solution to the puzzle of substrate specificity. Nat Struct Mol Biol 12: 835–838. 16205706 10.1038/nsmb1005-835

[24] Pavitt GD, Ramaiah KV, Kimball SR, Hinnebusch AG (1998) eIF2 independently binds two distinct eIF2B subcomplexes that catalyze and regulate guanine-nucleotide exchange. Genes Dev 12: 514–526. 9472020 10.1101/gad.12.4.514 PMC316533

[25] Dang Y, Kedersha N, Low WK, Romo D, Gorospe M, et al (2006) Eukaryotic initiation factor 2alpha-independent pathway of stress granule induction by the natural product pateamine A. J Biol Chem. 281: 32870–32878. 10.1074/jbc.M606149200 16951406

[26] Mazroui R, Sukarieh R, Bordeleau ME, Kaufman RJ, Northcote P, et al (2006) Inhibition of ribosome recruitment induces stress granule formation independently of eukaryotic initiation factor 2alpha phosphorylation. Mol Biol Cell 17: 4212–4219. 16870703 10.1091/mbc.E06-04-0318 PMC1635342

[27] Kramer S, Queiroz R, Ellis L, Webb H, Hoheisel JD, et al (2008) Heat shock causes a decrease in polysomes and the appearance of stress granules in trypanosomes independently of eIF2{alpha} phosphorylation at Thr169. J Cell Sci 121: 3002–3014. 18713834 10.1242/jcs.031823 PMC2871294

[28] Farny NG, Kedersha NL, Silver PA (2009) Metazoan stress granule assembly is mediated by P-eIF2alpha-dependent and -independent mechanisms. Rna 15: 1814–1821. 19661161 10.1261/rna.1684009 PMC2743051

[29] Emara MM, Ivanov P, Hickman T, Dawra N, Tisdale S, et al (2010) Angiogenin-induced tRNA-derived stress-induced RNAs promote stress-induced stress granule assembly. J Biol Chem 285: 10959–10968. 20129916 10.1074/jbc.M109.077560 PMC2856301

[30] Emara MM, Fujimura K, Sciaranghella D, Ivanova V, Ivanov P, et al (2012) Hydrogen peroxide induces stress granule formation independent of eIF2alpha phosphorylation. Biochem Biophys Res Commun 423: 763–769. 22705549 10.1016/j.bbrc.2012.06.033 PMC3399031

[31] Simpson CE, Ashe MP (2012) Adaptation to stress in yeast: to translate or not? Biochem Soc Trans 40: 794–799. 22817736 10.1042/BST20120078

[32] Brachmann CB, Davies A, Cost GJ, Caputo E, Li J, et al (1998) Designer deletion strains derived from Saccharomyces cerevisiae S288C: a useful set of strains and plasmids for PCR-mediated gene disruption and other applications. Yeast 14: 115–132. 9483801 10.1002/(SICI)1097-0061(19980130)14:2<115::AID-YEA204>3.0.CO;2-2

[33] Robinson JS, Klionsky DJ, Banta LM, Emr SD (1988) Protein sorting in Saccharomyces cerevisiae: isolation of mutants defective in the delivery and processing of multiple vacuolar hydrolases. Mol Cell Biol 8: 4936–4948. 3062374 10.1128/mcb.8.11.4936-4948.1988 PMC365587

[34] Sambrook J, Russell DW (2001) Molecular cloning: a laboratory manual (3rd ed.). Cold Spring Harbor, NY: Cold Spring Harbor Laboratory.

[35] Sambrook J, Fritsch EF, Maniatis T (1989) Molecular cloning : a laboratory manual. Cold Spring Harbor, N.Y.: Cold Spring Harbor Laboratory.

[36] Rothstein R (1991) Targeting, disruption, replacement, and allele rescue: integrative DNA transformation in yeast. Methods Enzymol 194: 281–301. 2005793 10.1016/0076-6879(91)94022-5

[37] Gueldener U, Heinisch J, Koehler GJ, Voss D, Hegemann JH (2002) A second set of loxP marker cassettes for Cre-mediated multiple gene knockouts in budding yeast. Nucleic Acids Res 30: e23. 11884642 10.1093/nar/30.6.e23 PMC101367

[38] Ter-Avanesyan MD, Dagkesamanskaya AR, Kushnirov VV, Smirnov VN (1994) The SUP35 omnipotent suppressor gene is involved in the maintenance of the non-Mendelian determinant [psi+] in the yeast Saccharomyces cerevisiae. Genetics 137: 671–676. 8088512 10.1093/genetics/137.3.671 PMC1206026

[39] Derkatch IL, Chernoff YO, Kushnirov VV, Inge-Vechtomov SG, Liebman SW (1996) Genesis and variability of [PSI] prion factors in Saccharomyces cerevisiae. Genetics 144: 1375–1386. 8978027 10.1093/genetics/144.4.1375 PMC1207691

[40] Gilks N, Kedersha N, Ayodele M, Shen L, Stoecklin G, et al (2004) Stress granule assembly is mediated by prion-like aggregation of TIA-1. Mol Biol Cell 15: 5383–5398. 15371533 10.1091/mbc.E04-08-0715 PMC532018

[41] Kimball SR, Horetsky RL, Ron D, Jefferson LS, Harding HP (2003) Mammalian stress granules represent sites of accumulation of stalled translation initiation complexes. Am J Physiol Cell Physiol 284: C273–284. 12388085 10.1152/ajpcell.00314.2002

[42] Anderson P, Kedersha N (2009) Stress granules. Curr Biol 19: R397–398. 19467203 10.1016/j.cub.2009.03.013

[43] Dori D, Choder M (2007) Conceptual modeling in systems biology fosters empirical findings: the mRNA lifecycle. PLoS One 2: e872. 17849002 10.1371/journal.pone.0000872 PMC1964809

[44] Alkalaeva EZ, Pisarev AV, Frolova LY, Kisselev LL, Pestova TV (2006) In vitro reconstitution of eukaryotic translation reveals cooperativity between release factors eRF1 and eRF3. Cell 125: 1125–1136. 16777602 10.1016/j.cell.2006.04.035

[45] Chakraburtty K (2001) Translational regulation by ABC systems. Res Microbiol 152: 391–399. 11421286 10.1016/s0923-2508(01)01210-4

[46] Kurata S, Nielsen KH, Mitchell SF, Lorsch JR, Kaji A, et al (2010) Ribosome recycling step in yeast cytoplasmic protein synthesis is catalyzed by eEF3 and ATP. Proc Natl Acad Sci U S A 107: 10854–10859. 20534490 10.1073/pnas.1006247107 PMC2890720

[47] Pisarev AV, Skabkin MA, Pisareva VP, Skabkina OV, Rakotondrafara AM, et al (2010) The role of ABCE1 in eukaryotic posttermination ribosomal recycling. Mol Cell 37: 196–210. 20122402 10.1016/j.molcel.2009.12.034 PMC2951834

[48] Scarcelli JJ, Viggiano S, Hodge CA, Heath CV, Amberg DC, et al (2008) Synthetic Genetic Array Analysis in Saccharomyces cerevisiae Provides Evidence for an Interaction Between RAT8/DBP5 and Genes Encoding P-Body Components. Genetics 179: 1945–1955. 18689878 10.1534/genetics.108.091256 PMC2516071

[49] Zou T, Rao JN, Liu L, Xiao L, Cui YH, et al (2012) Polyamines inhibit the assembly of stress granules in normal intestinal epithelial cells regulating apoptosis. Am J Physiol Cell Physiol 303: C102–111. 22555848 10.1152/ajpcell.00009.2012 PMC3404530

[50] Li CH, Ohn T, Ivanov P, Tisdale S, Anderson P (2010) eIF5A promotes translation elongation, polysome disassembly and stress granule assembly. PLoS One 5: e9942. 20376341 10.1371/journal.pone.0009942 PMC2848580

[51] Balagopal V, Parker R (2011) Stm1 modulates translation after 80S formation in Saccharomyces cerevisiae. RNA 17: 835–842. 21460238 10.1261/rna.2677311 PMC3078733

[52] Van Dyke N, Pickering BF, Van Dyke MW (2009) Stm1p alters the ribosome association of eukaryotic elongation factor 3 and affects translation elongation. Nucleic Acids Res 37: 6116–6125. 19666721 10.1093/nar/gkp645 PMC2764444

[53] Buchan JR, Parker R (2009) Eukaryotic stress granules: the ins and outs of translation. Mol Cell 36: 932–941. 20064460 10.1016/j.molcel.2009.11.020 PMC2813218

[54] Campbell SG, Hoyle NP, Ashe MP (2005) Dynamic cycling of eIF2 through a large eIF2B-containing cytoplasmic body: implications for translation control. J Cell Biol 170: 925–934. 16157703 10.1083/jcb.200503162 PMC2171431

[55] Campbell SG, Ashe MP (2006) Localization of the translational guanine nucleotide exchange factor eIF2B: a common theme for GEFs? Cell Cycle 5: 678–680. 16582624 10.4161/cc.5.7.2607

[56] Mortimer RK, Johnston JR (1986) Genealogy of principal strains of the yeast genetic stock center. Genetics 113: 35–43. 3519363 10.1093/genetics/113.1.35 PMC1202798

