# Supplementary figures and images for: Heat Shock-Induced Accumulation of Translation Elongation and Termination Factors Precedes Assembly of Stress Granules in S. cerevisiae
Source: PLoS One. 2013 Feb 25;8(2):e57083. doi: 10.1371/journal.pone.0057083 (PMC3581570; doi:10.1371/journal.pone.0057083)

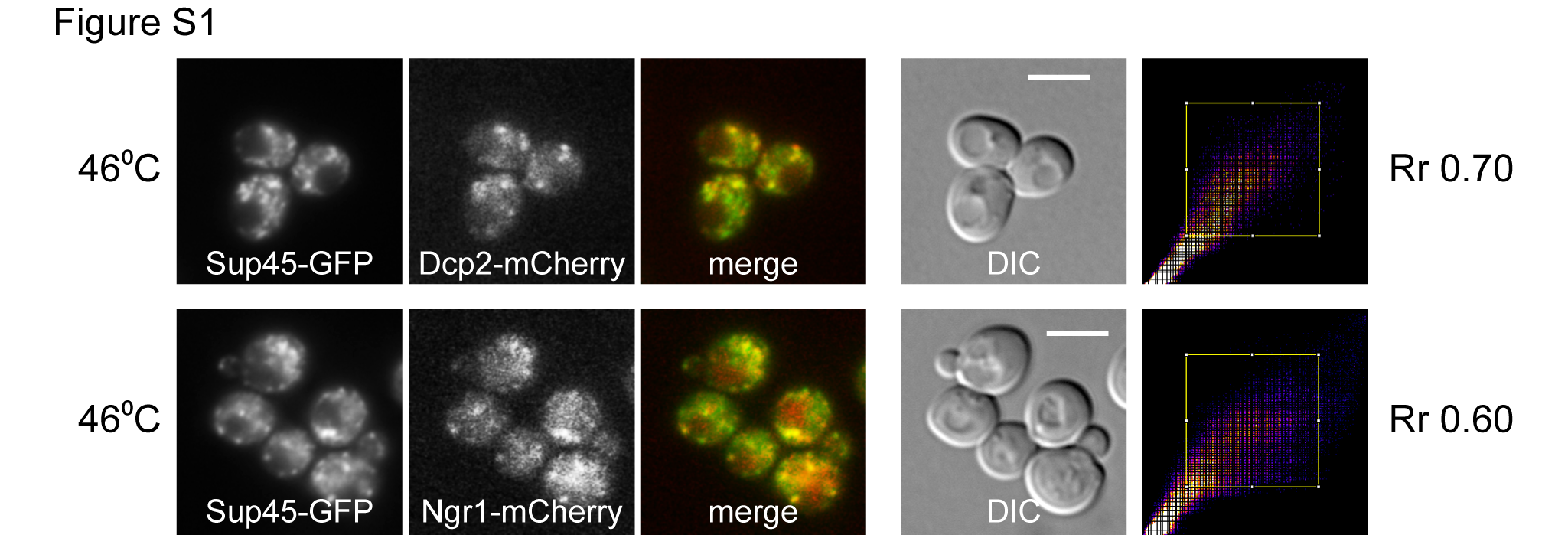

Supplement: Figure S1 — Factor eRF1 (Sup45p) co-localizes with Dcp2 and Ngr1 proteins in stress granules. (A) The subcellular distribution of Sup45-GFP and Dcp2-mCherry (CRY1636 strain) or (B) Sup45-GFP and Ngr1-mCherry (CRY1638) fusion proteins under heat shock at 46°C. Both pairs of proteins co-localize within stress granules. Values of the Pearsońs correlation coefficient (Rr) over 0.5 confirmed the co-localization. Scale bar 4 µm. (TIF) [file pone.0057083.s001.tif]

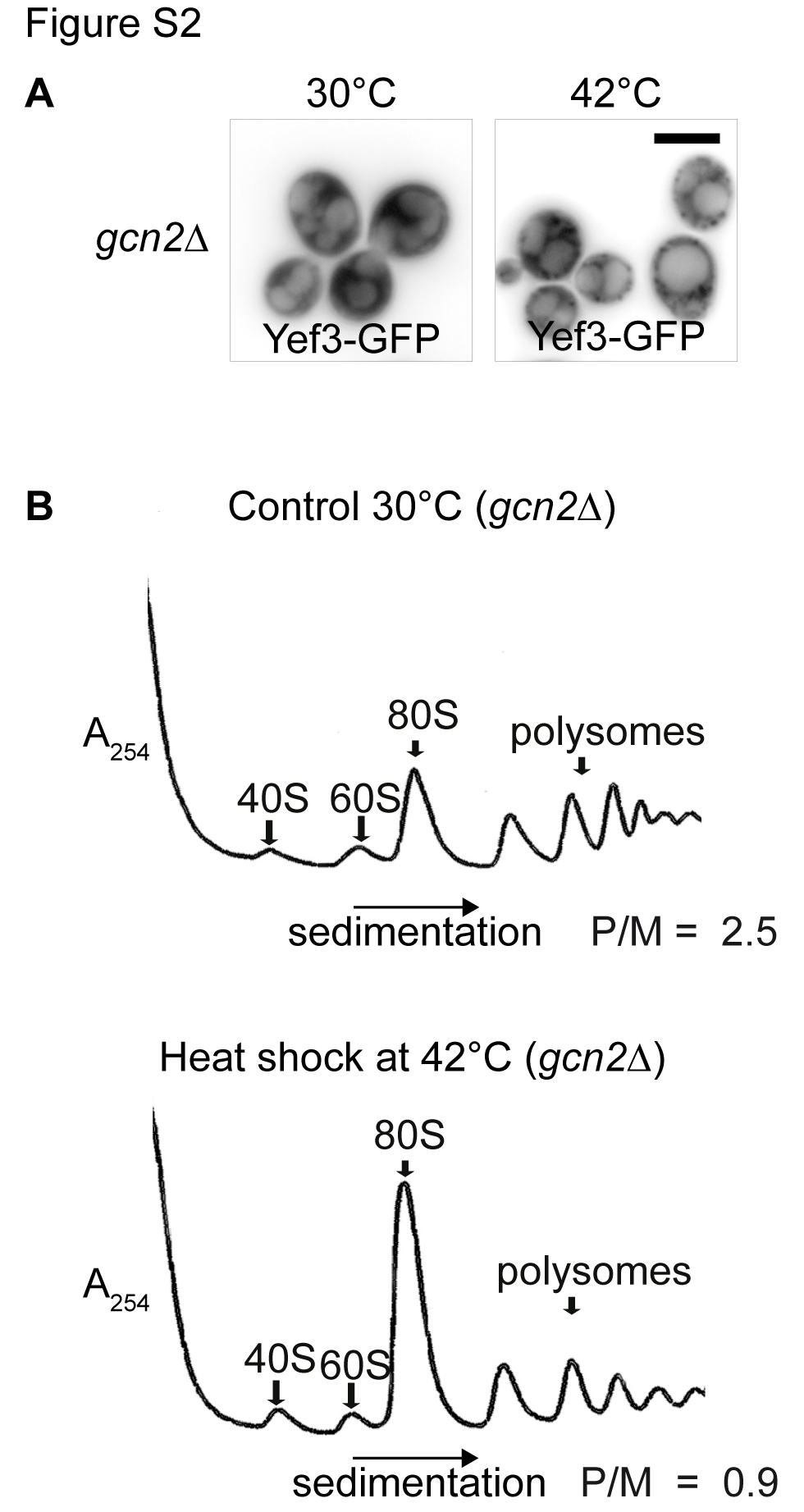

Supplement: Figure S2 — An accumulation of eEF3 factor and an alteration of polysome profile at 42°C are gcn2 Δ independent. (A) The distribution of Yef3-GFP protein in gcn2Δ strain (CRY1336 strain) at 30°C and under heat shock at 42°C. The protein accumulates in the same extend as the wild-type strain (compare with Figure 5) upon the heat shock. (B) Polysome profiles of gcn2Δ strain (CRY309 strain) at permissive temperature and after heat shock at 42°C for 10 minutes. Translation profile was altered in heat-shocked cells. P/M stands for polysome/monosome ratio. (TIF) [file pone.0057083.s002.tif]

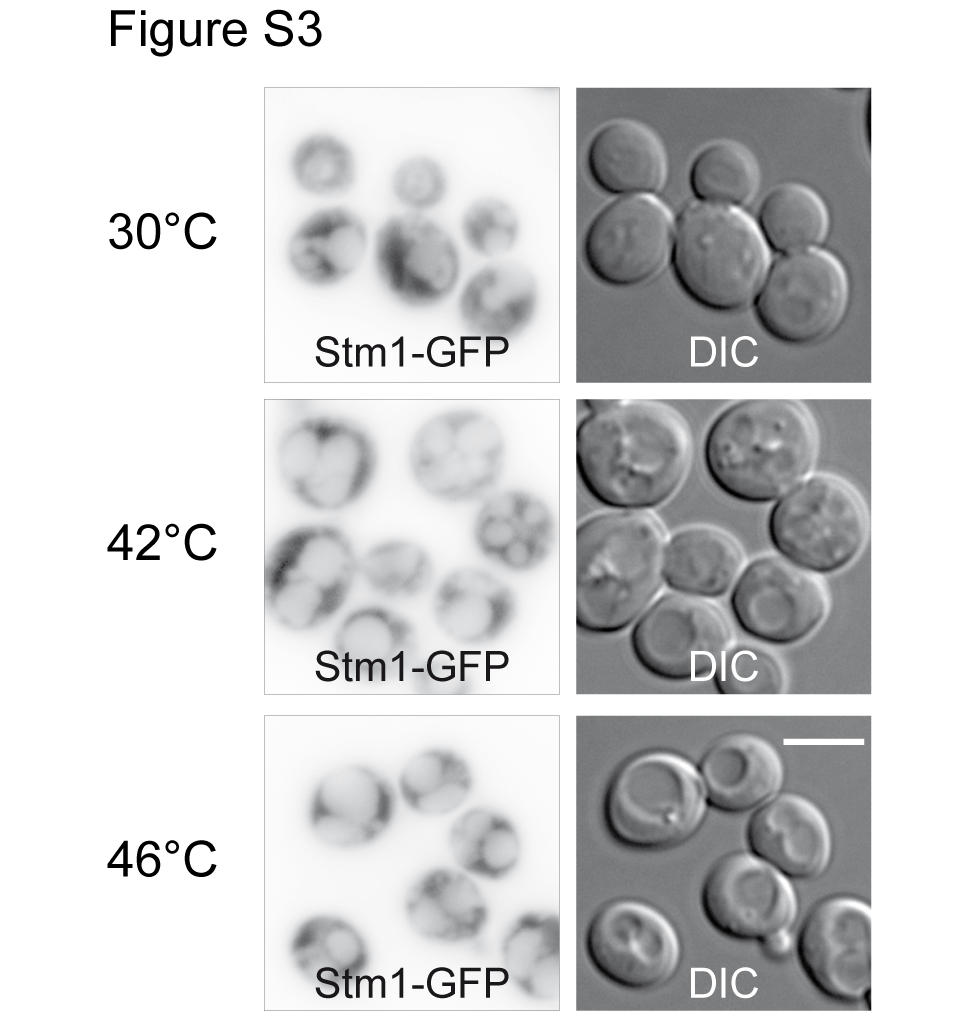

Supplement: Figure S3 — Subcellular distribution of Stm1-GFP fusion protein is not altered upon heat shock at 42°C and 46°C. The distribution of Stm1-GFP fusion protein (CRY1516 strain) remains diffusely cytosolic under all tested conditions. Scale bar 4 µm. (TIF) [file pone.0057083.s003.tif]

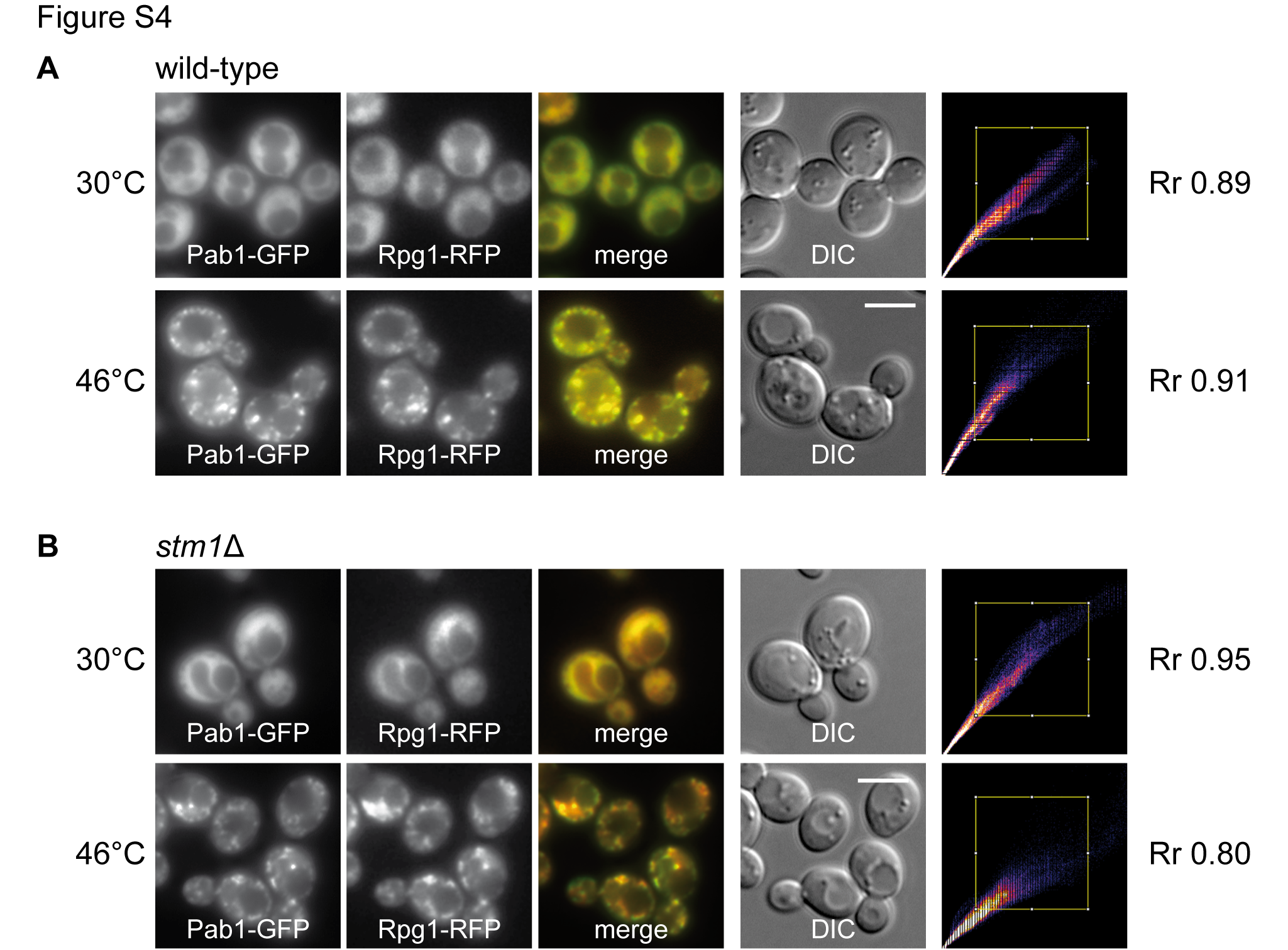

Supplement: Figure S4 — Deletion of the STM1 gene does not affect assembly of heat-induced SGs. (A) The distribution of Pab1-GFP and Rpg1-RFP proteins in wild-type cells (CRY528 strain) at 30°C and upon heat shock at 46°C for 10 minutes. Both proteins are accumulated in stress granules. (B) The stm1Δ mutant (CRY1760 strain) displayed a similar pattern of stress granules as the wild-type cells. Scale bar 4 µm. (TIF) [file pone.0057083.s004.tif]
